# Real-Time Imaging of the Intracellular Glutathione Redox Potential in the Malaria Parasite *Plasmodium falciparum*

**DOI:** 10.1371/journal.ppat.1003782

**Published:** 2013-12-05

**Authors:** Denis Kasozi, Franziska Mohring, Stefan Rahlfs, Andreas J. Meyer, Katja Becker

**Affiliations:** 1 Biochemistry and Molecular Biology, Interdisciplinary Research Center, Justus Liebig University Giessen, Giessen, Germany; 2 INRES-Chemical Signaling, University of Bonn, Bonn, Germany; The Hebrew University of Jerusalem, Israel

## Abstract

In the malaria parasite *Plasmodium falciparum*, the cellular redox potential influences signaling events, antioxidant defense, and mechanisms of drug action and resistance. Until now, the real-time determination of the redox potential in malaria parasites has been limited because conventional approaches disrupt sub-cellular integrity. Using a glutathione biosensor comprising human glutaredoxin-1 linked to a redox-sensitive green fluorescent protein (hGrx1-roGFP2), we systematically characterized basal values and drug-induced changes in the cytosolic glutathione-dependent redox potential (*E*_GSH_) of drug-sensitive (3D7) and resistant (Dd2) *P. falciparum* parasites. Via confocal microscopy, we demonstrated that hGrx1-roGFP2 rapidly detects *E*_GSH_ changes induced by oxidative and nitrosative stress. The cytosolic basal *E*_GSH_ of 3D7 and Dd2 were estimated to be −314.2±3.1 mV and −313.9±3.4 mV, respectively, which is indicative of a highly reducing compartment. We furthermore monitored short-, medium-, and long-term changes in *E*_GSH_ after incubation with various redox-active compounds and antimalarial drugs. Interestingly, the redox cyclers methylene blue and pyocyanin rapidly changed the fluorescence ratio of hGrx1-roGFP2 in the cytosol of *P. falciparum*, which can, however, partially be explained by a direct interaction with the probe. In contrast, quinoline and artemisinin-based antimalarial drugs showed strong effects on the parasites' *E*_GSH_ after longer incubation times (24 h). As tested for various conditions, these effects were accompanied by a drop in total glutathione concentrations determined in parallel with alternative methods. Notably, the effects were generally more pronounced in the chloroquine-sensitive 3D7 strain than in the resistant Dd2 strain. Based on these results hGrx1-roGFP2 can be recommended as a reliable and specific biosensor for real-time spatiotemporal monitoring of the intracellular *E*_GSH_ in *P. falciparum*. Applying this technique in further studies will enhance our understanding of redox regulation and mechanisms of drug action and resistance in *Plasmodium* and might also stimulate redox research in other pathogens.

## Author Summary

In the malaria parasite *Plasmodium falciparum*, cellular redox reactions play important roles not only in redox-regulatory processes and antioxidant defense but also in the mechanisms of drug action and drug resistance. The tripeptide glutathione is present in malaria parasites in millimolar concentrations and represents the most important low molecular weight antioxidant. In this work, we describe the application of a genetically encoded glutathione biosensor comprising human glutaredoxin-1 linked to a redox-sensitive green fluorescent protein. By targeting this probe to the parasites' cytosol, we were able to verify its stability and responsiveness to the redox environment. Using this probe in living parasites, we further studied the effects of various redox active compounds and antimalarial drugs on the cytosolic glutathione redox potential in short, medium, and long-term experiments. Interestingly, after 24 h most antimalarial drugs, including quinolines and artemisinin derivatives, induced pronounced changes in the redox potential in pharmacologically meaningful concentrations. The chloroquine-sensitive *P. falciparum* 3D7 strain was generally more susceptible to these changes than the resistant Dd2 strain. We propose the hGrx1-roGFP2 probe as a reliable and most valuable tool for studying redox metabolism in living malaria parasites.

## Introduction

Malaria is a major disease burden in 106 countries, causing an estimated 225 million cases and, as reported for 2010, 665,000 to 1,133,000 human deaths each year in tropical and sub-tropical regions [1]. Despite recent progress [2], drug resistance remains a major obstacle to the effective and sustainable control of malaria. Cellular redox reactions play important roles not only in redox regulatory processes and antioxidant defense but also in the mechanisms of drug action and drug resistance in malaria parasites [3], [4]. Recent advances in parasite biology suggest a compartmentalization of redox metabolism [5] as well as of cellular processes that are sites of drug action [6].

Indeed, several antimalarial drugs have been reported to act through the induction of oxidative [7] or nitrosative stress [8]. Artemisinin (ART) derivatives, e.g., are activated via reductive cleavage of their peroxide bond by intracellular iron (Fe^2+^) or heme –generating, carbon-centered free radicals [9], [10] with the potential to alkylate vital cellular components including the tripeptide glutathione (γ-Glu-Cys-Gly, GSH). Methylene blue (MB), quinoline [11], and artemisinin-based [6] antimalarial drugs have been shown to accumulate in the food vacuole and inhibit the formation of hemozoin [12]. Non-crystallized toxic heme exits the food vacuole and is degraded in the cytosol with GSH as a cofactor. This process is inhibited by chloroquine (CQ) and amodiaquine (AQ) [13], leading to an intensive discussion about the role of oxidative stress in the mechanism of action of 4-aminoquinolines [14], [15], [16]. Furthermore, MB is known to be a redox cycler and a substrate and inhibitor of glutathione reductase (PfGR) in the cytosol [17], [18], [19], and the mutant *P. falciparum* chloroquine resistance transporter (PfCRT) may also be involved in the transport of glutathione [20]. Hence, redox metabolism seems to play an important role in antimalarial drug action and resistance and deserves to be studied in more detail.

The *P. falciparum* glutathione redox system comprises the electron donor NADPH, a highly active PfGR located in the cytosol and the apicoplast, and reduced/oxidized glutathione (GSH/GSSG) [21], [22], [23]. Glutathione levels have been shown to be regulated via glutathione biosynthesis, glutathione efflux, and reduction via GR. *De novo*, GSH is sequentially synthesized in the cytosol by γ-glutamylcysteine synthetase (γ-GCS) and glutathione synthase. The GSH/GSSG redox couple is the major redox buffer in the cytosol [21], [22] and functions as an indicator of the cellular redox status and oxidative stress [24]. In malaria parasites, GSH is a key player in the detoxification of reactive oxygen (ROS) and nitrogen species (RNS), which are produced by antimalarial drugs, hemoglobin digestion, and the host's immune system [25], [26]. The midpoint redox potential of the GSH:GSSG redox couple (*E^0^*'_GSH_) at pH 7.0, physiologic ionic strength and 25°C is −240 mV [24]. Importantly, changes in *E*_GSH_ appear to correlate with the biological status of cells including proliferation (−240 mV), differentiation (−200 mV), and apoptosis (−170 mV) [24]. Using global values (2.39 mM GSH; 8.4 µM GSSG [27]), the *E*_GSH_ for the *Plasmodium* trophozoite cytosol at pH 7.2 and 37°C has so far only been roughly estimated [22].

Conventionally, GSSG and GSH are measured via reverse-phase high-performance liquid chromatography or enzymatically via glutathione reductase-dependent reactions and the 5,5′-dithiobis(2-nitrobenzoic acid) (also known as DTNB or Ellman's reagent) recycling assay [28]. However, due to the disruption of organelle/cell integrity followed by a mixing of compartments and oxidation of GSH to GSSG, such estimates lack sub-cellular relevance [28], and dynamic measurements are impossible. In living cells, other thiol-reactive dyes can be applied to assess redox homeostasis, this is, however, usually limited to detecting cellular levels of e.g. reduced glutathione [29].

To overcome the limitations of these redox potential measurements, genetically encoded biosensors have been developed, including redox-sensitive green fluorescent protein (roGFP) and yellow fluorescent protein (YFP) [30], [31], which enable non-invasive and dynamic redox measurements *in vivo*. Recently, a highly specific and sensitive glutathione biosensor consisting of human glutaredoxin-1 (hGrx1) fused to roGFP2 was described [32]. Interestingly, hGrx1-roGFP2 was reported to detect nanomolar changes in GSSG concentrations against a backdrop of millimolar concentrations of reduced GSH on a scale of seconds to minutes [32]. This detection method is based on the ratiometric imaging of the hGrx1-roGFP2 sensor by using two different excitation wavelengths (405 nm and 488 nm) and fluorescence measurements in the green channel (500–530 nm). Transition from reduction to oxidation changes the state of the disulfide bond in the redox sensor, increases the fluorescence intensity of the 405 nm emission peak, and decreases the intensity in the 488 nm peak. This allows one to measure the *E*_GSH_ independently from different expression levels of hGrx1-roGFP2, photo bleaching, or autofluorescence in the cells [28].

As described here, we successfully transfected CQ-sensitive and resistant *P. falciparum* parasites with the hGrx1-roGFP2 probe and characterized the functionality of the redox sensor. Furthermore we provide the first data on the effects of antimalarial drugs on the cytosolic glutathione redox potential in *Plasmodium* using the redox probe in combination with confocal live cell imaging. Based on our data we propose hGrx1-roGFP2 as a suitable and powerful tool to study redox-related changes in malaria parasites.

## Results

### hGrx1-roGFP2 as a sensor of the glutathione redox potential in *P. falciparum*

**Figure 1:**
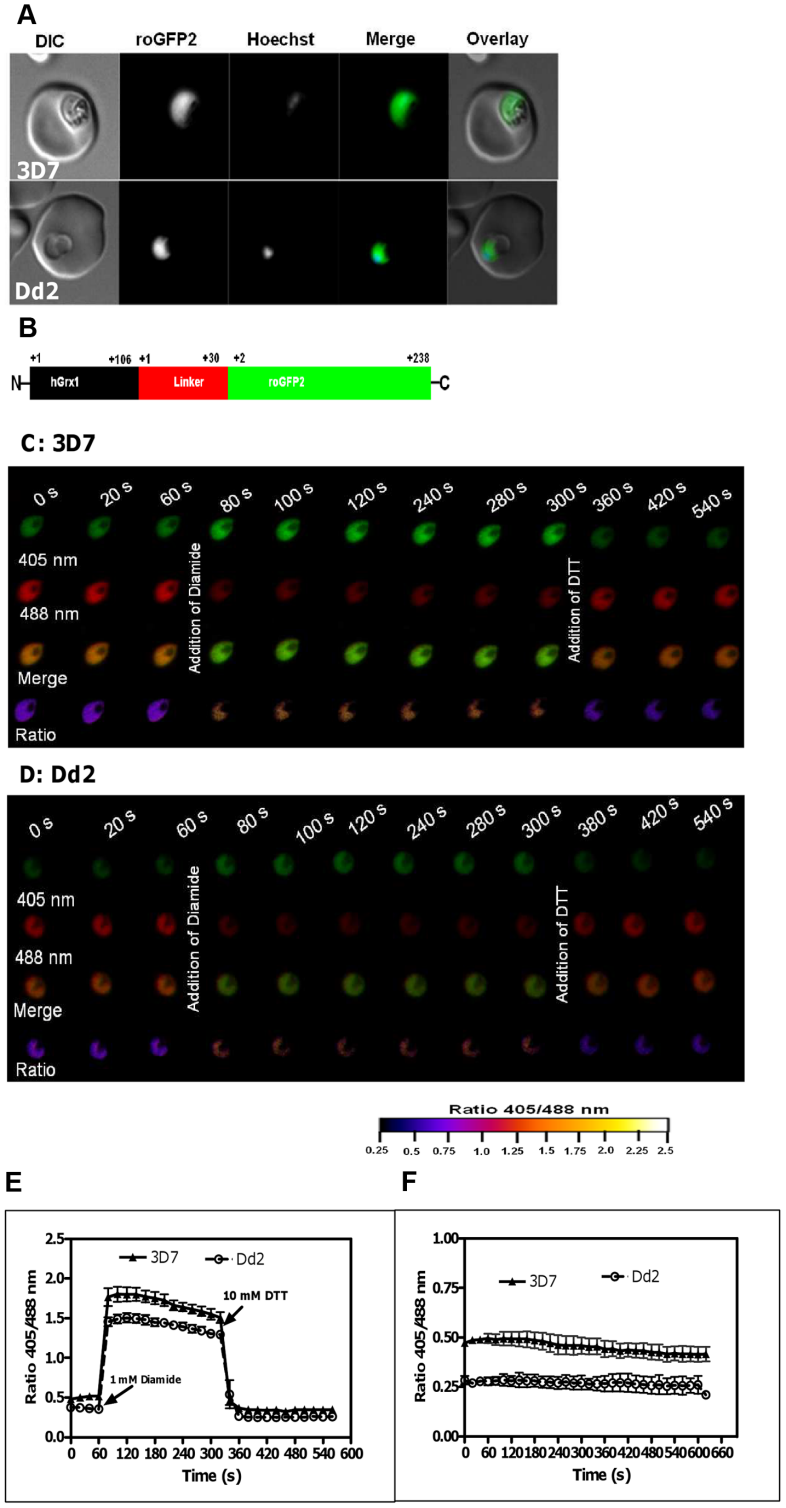
Real-time imaging of the glutathione redox potential in *P. falciparum*. **A.** Confocal live cell images of 3D7 and Dd2 parasites showing the expression of hGrx1-roGFP2 localized in the cytosol. **B**. hGrx1-roGFP2 is a fusion protein with human glutaredoxin 1 (hGrx1, black) fused to the N-terminal end of roGFP2 (green) through a linker (red) comprising 30 amino acids (Gly-Gly-Ser-Gly-Gly)_6_. Live cell imaging of the trophozoite stages of (**C**) 3D7^hGrx1-roGFP2^ and (**D**) Dd2^hGrx1-roGFP2^. After 60 s, 3D7 (**C**) and Dd2 (**D**) parasites were treated with 1 mM diamide followed 4 min later by addition of 10 mM dithiothreitol (DTT). 405 nm, 488 nm, merge (405/488 nm), and false color ratio images at different time points are shown. **E**. The ratio of emissions (ratio 405/488 nm) after excitation at 405 and 488 nm was computed for both strains and plotted against time. Data from 5 trophozoites for each strain were analyzed per data point. **F**. Fluorescence ratio as a function of time. The ratio 405/488 nm remained stable over a period of 10 min. Data from 5 trophozoites for each strain were analyzed per data point. Mean and standard errors of the mean are shown.

The reliable responsiveness of the Grx-roGFP probe to various GSH/GSSG ratios and GSH concentrations has been investigated in great detail and verified in previous studies [31], [32], [33]. To gain insight into the redox-sensing properties of hGrx1-roGFP2 [32] in *P. falciparum*, we transfected the hGrx1-roGFP2 gene cloned into the pARL-1a+ expression vector [34] into the CQ-sensitive 3D7 and the CQ-resistant Dd2 strains of the parasite. Following 3–4 weeks of selection with 2 nM WR99210, the stable transfectants showed a strong hGrx1-roGFP2 fluorescence signal in the parasite's cytosol when visualized by confocal live cell microscopy (Fig. 1A). Furthermore, we confirmed the expression of the full-length fusion hGrx1-roGFP2 protein with the predicted size of 47 kDa in parasite lysates of 3D7 (3D7^hGrx1-roGFP2^) and Dd2 (Dd2^hGrx1-roGFP2^) strains via western blotting (Fig. S1). The hGrx1-roGFP2 protein was also constantly and comparably expressed in parasites exposed to different treatment regimes as shown for different concentrations of diamide, methylene blue, and artemisinin via western blotting (Fig. S1). A tendency towards higher hGrx1-roGFP2 expression was observed for the 24 h incubations with low drug concentrations. This is, however, unlikely to affect the signal provided by the probe, which is based on ratiometry [28]. Furthermore, as also demonstrated with anti-GFP and anti-hGrx antibodies, no degradation of the redox sensor was observed under the experimental conditions chosen. Only when incubating with high concentrations of the stressor diamide (≥1 mM), which led to the destruction of cells and a loss of protein, a degradation of hGrx1-roGFP2 was detected (as shown with the anti-hGrx antibody) (Fig. S1).

Since the roGFP-biosensor is operating on the basis of a ratiometric measurement, potential quenching of the signals is not problematic as long as they are strong enough to be measured [28]. However, in order to exclude a disturbing influence of the autofluorescence of hemoglobin in our experimental system, we determined the autofluorescence of non-infected RBCs. Indeed, the autofluorescence was negligible (<2%) and did not interfere with the redox ratio values of hGrx-roGFP in parasitized cells (Fig. S2).

Next, we excited both parasite strains subsequently at 405 nm and 488 nm, and the ratio of emissions (fluorescence ratio 405/488 nm) in the green channel (500–530 nm) was calculated. Our data indicate that a 1 min treatment with 1 mM diamide caused maximum oxidation of hGrx1-roGFP2 in 3D7^hGrx1-roGFP2^ (Figs. S3A–C) and in Dd2^hGrx1-roGFP2^ (Figs. S3D–F). Similar to trophozoite stages, 1 mM diamide fully oxidized hGrx1-roGFP2 in schizonts (Fig. S4A) and gametocytes (Fig. S4B). On the other hand, 10 mM dithiothreitol (DTT) fully reduced hGrx1-roGFP2 in both strains – as shown for 3D7 trophozoite stages in Fig. S4C. Since hGrx1-roGFP2 is already fully reduced in the control cells, there was little change after DTT treatment.

To validate the use of hGrx1-roGFP2 for imaging dynamic changes in *E*_GSH_ in malaria parasites, we treated both transfected strains sequentially, first with 1 mM diamide and 4 min later with 10 mM DTT. In this experimental series, within seconds after adding 1 mM diamide, the fluorescence ratio 405/488 nm increased from 0.50±0.02 to 1.79±0.04 in 3D7^hGrx1-roGFP2^ and from 0.37±0.01 to 1.49±0.03 in Dd2^hGrx1-roGFP2^, indicating rapid oxidation of hGrx1-roGFP2 (Figs. 1C–E). Subsequently, after adding 10 mM DTT, the fluorescence ratio 405/488 nm decreased again to 0.34±0.01 and 0.26±0.01 in 3D7^hGrx1-roGFP2^ and Dd2^hGrx1-roGFP2^, respectively, indicating a reduction of hGrx1-roGFP2 (Figs. 1C–E).

### The cytosolic basal glutathione redox potential is strongly reducing in *P. falciparum*

In order to study the stability of the cytosolic basal E_GSH_ we monitored the basal fluorescence ratios 405/488 nm of untreated 3D7^hGrx1-roGFP2^ and Dd2^hGrx1-roGFP2^ for an extended period of time (Fig. 1F). For at least 30 min, the ratios were found to be constant in the cells under the microscope. Our short-term experiments did not usually take longer than 5 min. However, remarkably, the basal fluorescence ratios 405/488 nm were found to be significantly different for 3D7^hGrx1-roGFP2^ and Dd2^hGrx1-roGFP2^, with values of 0.59±0.09 (n = 30) and 0.29±0.08 (n = 30), respectively (n = 30 corresponds to 30 independent measurements on cells from different experimental series and days). The 3D7 strain constantly showed a higher ratio than the Dd2 strain. This observation might at least partially be explained by higher concentrations of total glutathione in Dd2 (approx. 2-fold [21]), which induce a stronger basal reduction of hGrx1-roGFP2 in Dd2.

In order to estimate the basal *E*_GSH_ in the cytosol of *P. falciparum*, we computed the degree of oxidation (OxD_roGFP2_) from the fluorescence intensity measured in the two transfected parasite strains at the resting state (basal), after maximal oxidation (1 mM diamide) and after full reduction (10 mM DTT). As described [31], [33], we used a midpoint redox potential of roGFP2 of −280 mV [31] and a consensus cytosolic pH = 7.20 [22] at a temperature of 37.0°C for our calculations. According to this approach, the basal cytosolic *E*_GSH_ of 3D7 and Dd2 were −314.2±3.1 mV and −313.9±3.4 mV, respectively.

### The dynamic range of hGrx1-roGFP2 is higher in *P. falciparum* 3D7 than in Dd2

The dynamic range is the ratio between the highest and the lowest 405/488 nm ratio, i.e., the range between the completely reduced and the completely oxidized state of the cell. Previously, the dynamic range of roGFP2 had been reported to differ between different cellular compartments [35]. In order to determine the dynamic range of hGrx1-roGFP2 in *P. falciparum*, we divided the average highest fluorescence ratio 405/488 nm of the diamide time course by the average lowest ratio in the DTT time course of both strains. Based on this, the dynamic range of hGrx1-roGFP2 was determined to be 6.36±0.73 in the 3D7 strain and 5.28±0.49 in Dd2.

### Oxidative and nitrosative stress affect the glutathione redox potential

**Figure 2:**
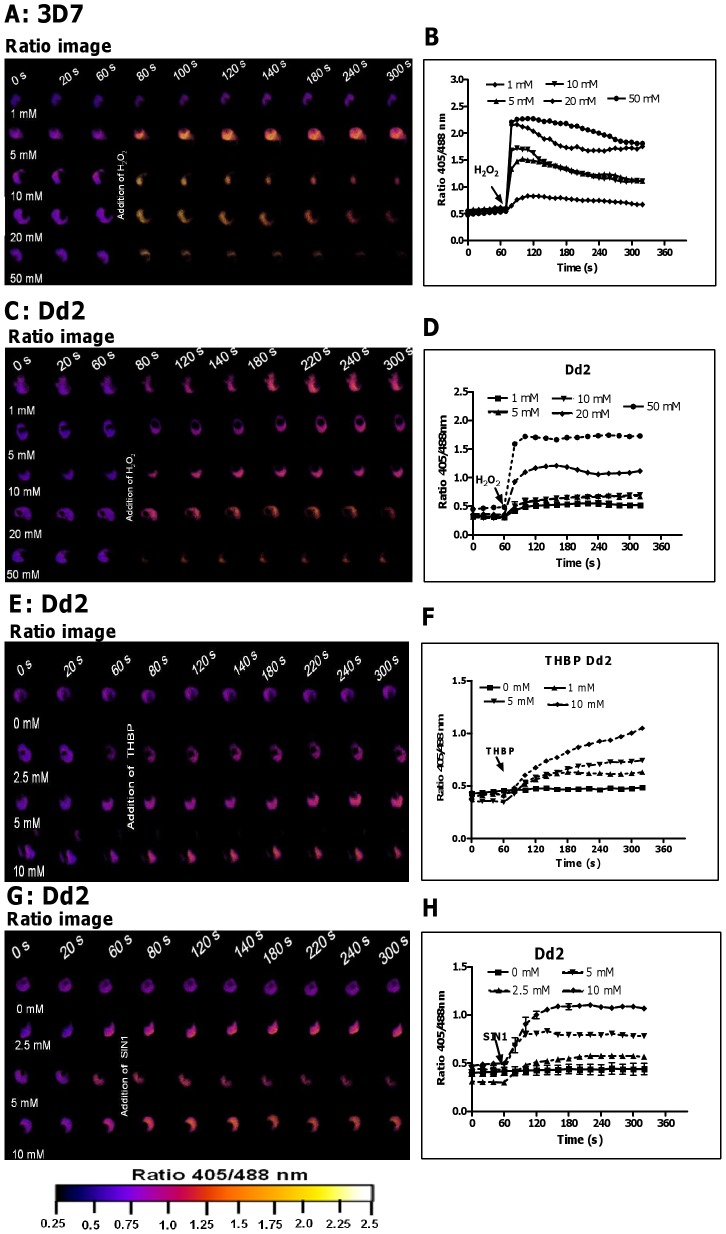
hGrx1-roGFP2 is suitable for monitoring the effects of oxidative and nitrosative stress on the glutathione redox potential. After 60*P. falciparum* parasites expressing hGrx1-roGFP2 were treated with different concentrations of H_2_O_2_ (**A**, **B**: 3D7; **C**, **D**: Dd2), *tert*-butyl hydroperoxide (TBHP: **E**, **F**: Dd2) and 3-morpholinosydnonimine hydrochloride (SIN1: **G**, **H**: Dd2) and monitored for 4 min. Ratio images (405/488 nm) of the cells at different time points are provided (**A**, **C**, **E**, **G**). Furthermore, the ratios 405/488 nm were computed and plotted against time (**B**, **D**, **F**, **H**). For each concentration, data from 3 trophozoites were analyzed per data point. Mean and standard errors of the mean are shown.

To investigate whether hGrx1-roGFP2 is suitable for monitoring changes in *E*_GSH_ in *P. falciparum* after oxidative stress, we treated 3D7 and Dd2 trophozoites with H_2_O_2_ or *tert*-butyl hydroperoxide (TBHP). Following treatment of 3D7^hGrx1-roGFP2^ (Fig. 2A, B) and Dd2^hGrx1-roGFP2^ (Fig. 2C, D) with 1 mM H_2_O_2_, a rapid increase in the fluorescence ratio 405/488 nm (on a scale of seconds) was observed, but much higher concentrations (50 mM) were required to attain full oxidation. At the same concentrations of H_2_O_2_ (e.g. 10 mM H_2_O_2_), a stronger oxidation was observed in 3D7 (Figs. 2A, B) than in Dd2 (Figs. 2C, D). TBHP is frequently used instead of H_2_O_2_ as an oxidant since it is not a substrate for catalase. Treatment of *P. falciparum*, which does not possess a genuine catalase but rather a number of thioredoxin-dependent peroxidases, with TBHP resulted in a rapid increase in the fluorescence ratio 405/488 nm (Figs. 2E, F), which was more pronounced than the oxidation caused by equal concentrations of H_2_O_2_ (Figs. 2C, D).

In order to induce nitrosative stress, we used 3-morpholinosydnonimine hydrochloride (SIN-1; a peroxynitrite generator). SIN1 (Fig. 2G, H) also rapidly increased the fluorescence ratio 405/488 nm in the Dd2 strain.

### Direct interactions of antimalarial drugs and redox-active agents with isolated hGrx1-roGFP2

Before testing the short-term and long-term effects of antimalarial compounds on the GSH-dependent redox potential of malaria parasites, we characterized their direct *in vitro* interaction with recombinant hGrx1-roGFP2 in the absence of GSH. This was important for enabling differentiation between direct interactions of the compounds with the probe (background signals) and pharmacological effects of the compounds on the glutathione redox potential.

**Figure 3:**
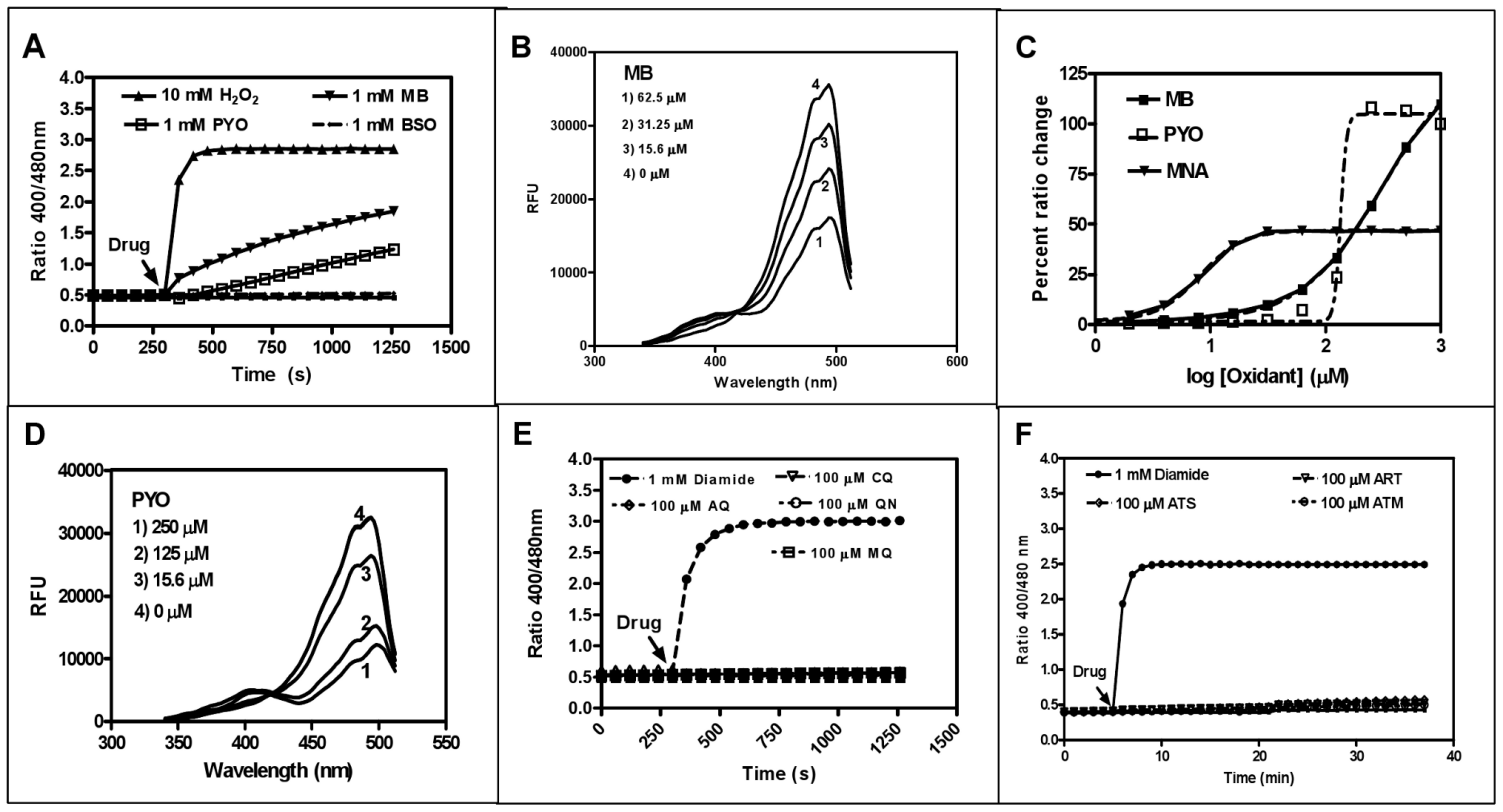
*In vitro* interaction of recombinant hGrx1-roGFP2 with antimalarial drugs. **A**. Oxidation of reduced hGrx1-roGFP2 by methylene blue (MB) and pyocyanin (PYO) at 1 mM in comparison to 10 mM H_2_O_2_ and 1 mM BSO. **B**. Excitation spectra of hGrx1-roGFP2 with different concentrations of MB. **C**. Ratio change of hGrx1-roGFP2 plotted against concentrations of MB, PYO, and MNA. **D**. Excitation spectra of hGrx1-roGFP2 with different concentrations of PYO. **E**. Oxidation of reduced hGrx1-roGFP2 with quinoline and (**F**) artemisinin-based antimalarial drugs at 100 µM.

The tested drugs included methylene blue (MB), quinoline drugs (chloroquine (CQ), amodiaquine (AQ), quinine (QN), mefloquine (MQ), artemisinin derivatives (artemisinin (ART), artesunate (ATS), artemether (ATM), as well as pyocyanin (PYO), an analog of MB that has anti-plasmodial activity [36], menadione (MNA), a naphthoquinone antimalarial lead compound [37], and a number of other standard redox-active compounds. Table 1 and Figure 3 summarize the changes in the fluorescence ratio 405/488 nm of the recombinant hGrx1-roGFP2 protein induced by the compounds in different concentrations and at different incubation times.

**Table 1:** Effects of redox-active compounds and antimalarial drugs on the redox ratio of isolated recombinant hGrx1-roGFP2 *in vitro* and IC_50_ values of the antimalarial drugs on the *P. falciparum* strains 3D7 and Dd2.

Drugs/redox-active compounds (abbreviations)	Increase in fluorescence ratio of isolated hGrx1-roGFP2^a^	Fold change in fluorescence ratio of isolated hGrx1-roGFP2^b^	IC_50_ on *P. falciparum* 3D7 [nM]^c^	IC_50_ on *P. falciparum* Dd2 [nM]^c^
Diamide (control) (DMD)	0.49→3.06 (1 mM, 5 min)	6.24 (1 mM, 5 min)	nd	nd
Glutathione disulfide (GSSG)	0.49→3.20 (1 mM, 5 min)	6.54 (1 mM, 5 min)	nd	nd
**ROS generators**				
Hydrogen peroxide (H_2_O_2_)	No major effect (100 µM, 5 min) 0.49→3.21 (10 mM, 5 min)	No major effect (100 µM, 5 min) 6.56 (10 mM, 5 min)	nd	nd
*tert*-Butyl hydroperoxide (TBHP)	0.49→0.57 (10 mM, 5 min)	1.16 (10 mM, 5 min)	nd	nd
2,2′-Azobis-2-methyl-propanimidamide dihydrochloride (AAPH)	0.49→1.65 (10 mM, 1 h)	3.36 (10 mM, 1 h)	nd	nd
Paraquat (PQT)	0.49→0.53 (10 mM, 5 min)	1.09 (10 mM, 5 min)	45400	21000
**RNS donors**				
Sodium nitroprusside (SNP)	0.49→1.65 (1 mM, 10 min)	3.36 (1 mM, 10 min)	6000	7400
3-Morpholino-sydnonimine hydrochloride (SIN1)	0.49→1.52 (10 mM, 10 min)	3.11 (10 mM, 10 min)	nd	nd
**Redox cyclers, inhibitors of GSH synthesis**				
Methylene blue (MB)	0.49→1.69 (1 mM, 15 min) 0.49→7.35 (1 mM, 24 h)	3.44 (1 mM, 15 min) 7.35 (1 mM, 24 h)	3.3	5.3
Pyocyanin (PYO)	0.49→3.13 (1 mM, 24 h)	6.39 (1 mM, 24 h)	58	195
Menadione (MNA)	0.49→1.03 (1 mM, 15 min)	2.10 (1 mM, 15 min)	nd	nd
L-Buthionine sulfoximine (BSO)	0.49→0.49 (1 mM, 24 h)	1.01 (1 mM, 24 h)	26 300	58900
**Quinoline drugs**				
Chloroquine (CQ)	0.49→0.58 (100 µM, 24 h)	1.19 (100 µM, 24 h)	8.6	90.2
Amodiaquine (AQ)	0.49→0.49 (100 µM, 24 h)	1.01 (100 µM, 24 h)	18.6	7.2
Quinine (QN)	0.49→0.60 (100 µM, 24 h)	1.23 (100 µM, 24 h)	210	136
Mefloquine (MQ)	0.49→0.49 (100 µM, 24 h)	1.01 (100 µM, 24 h)	8.0	19.5
**Artemisinin derivatives**				
Artemisinin (ART)	0.49→0.68 (100 µM, 24 h	1.39 (100 µM, 24 h)	17.3	20.4
Artesunate (ATS)	0.49→0.63 (100 µM, 24 h	1.29 (100 µM, 24 h)	4.4	5.2
Artemether (ATM)	0.49→0.70 (100 µM, 24 h)	1.42 (100 µM, 24 h)	5.9	8.4

a In this column the absolute change in the fluorescence ratio 405/488 nm of isolated recombinant hGrx1-roGFP2 after incubation with the compounds at given concentrations and time points is shown. The ratio 405/488 nm of reduced recombinant hGrx1-roGFP2, which served as starting point for the experiments, was 0.49±0.01.

b In this column the fold change in the fluorescence ratio 405/488 nm of isolated recombinant hGrx1-roGFP2 after incubation with the compounds at given concentrations and time points is shown.

c Please see also references [21], [36], [38], and [67] for details on IC_50_-values.

The redox cycler MB caused a pronounced and immediate increase in the fluorescence ratio 405/488 nm (Fig. 3A) as well as spectral changes (Fig. 3B) in recombinant hGrx1-roGFP2. Like MB, PYO [36] and MNA [37] are active as redox-cycling agents. Notably, MB and PYO but not MNA (Fig. 3C) were able to fully oxidize recombinant hGrx1-roGFP2 after 24 h of incubation to ratios comparable to the changes induced by GSSG, diamide, and H_2_O_2_ after 5 min (Fig. S5A). The partial oxidation of recombinant roGFP2 by MNA (even after 24 h of incubation) is in agreement with previous reports [31]. The changes induced in the hGrx1-roGFP2 spectrum by MB (Fig. 3B) and PYO (Fig. 3D) were similar in shape and intensity to those caused by diamide (Fig. S5B), GSSG, H_2_O_2_, SIN1, and AAPH (Figs. S5C–F) as shown by plotting the fluorescence intensities, measured as relative fluorescence units (RFU) as a function of wavelength at the respective drug concentrations.

Other antimalarial drugs including the quinolines CQ, AQ, MQ, or QN (Fig. 3E) and artemisinin derivatives such as ART, ATS, and ATM (Fig. 3F) did not cause a major increase in the fluorescence ratio of recombinant hGrx1-roGFP2, even at concentrations up to 100 µM and after 24 h incubation.

### Short-term effects of antimalarial drugs and redox-active compounds on the cytosolic redox potential in *Plasmodium*

**Figure 4:**
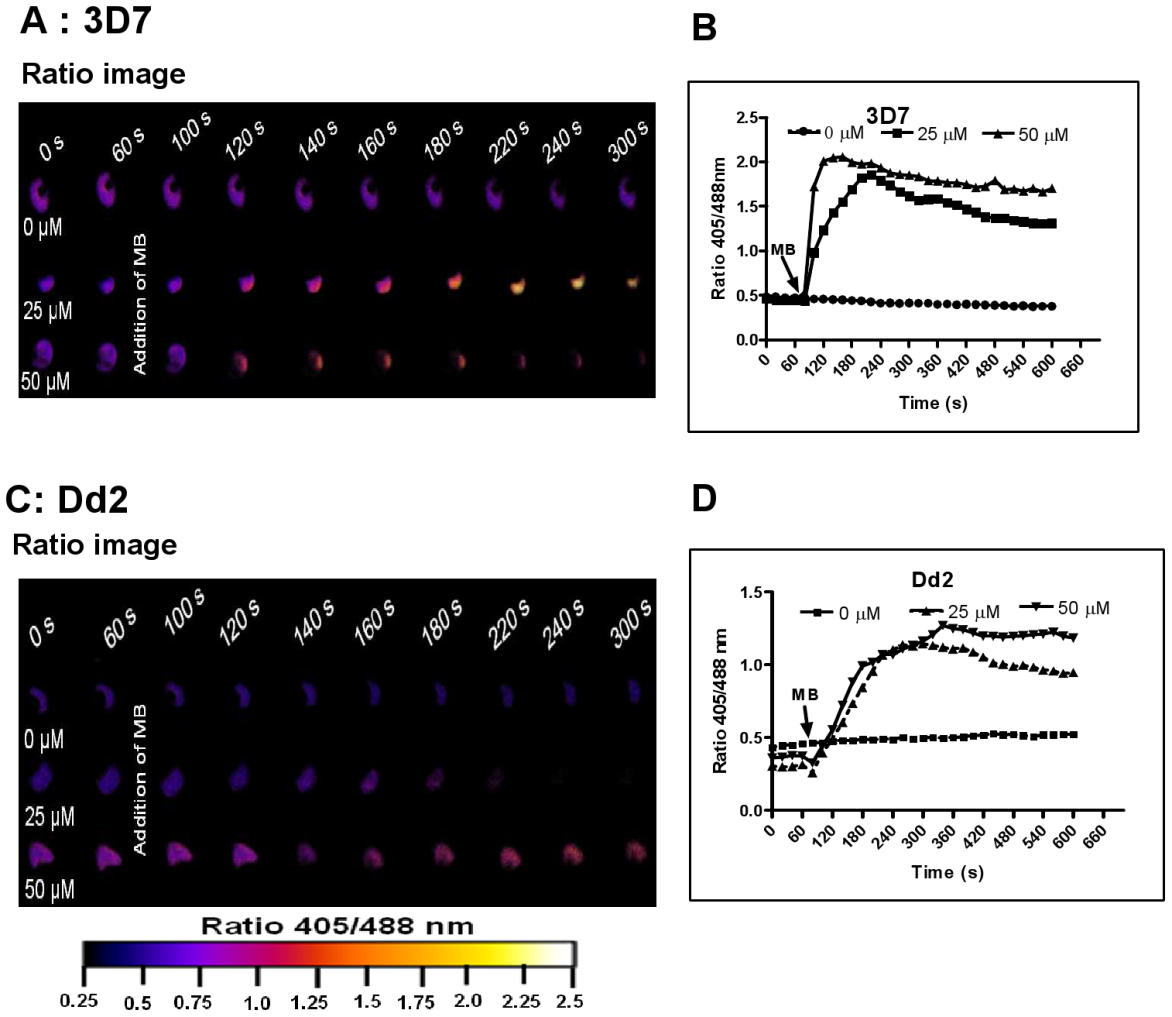
Immediate oxidation of the *P. falciparum* cytosol by methylene blue. Different concentrations of methylene blue (MB) were added to 3D7 (**A**, **B**) and Dd2 (**C**, **D**) trophozoite stage parasites of *P. falciparum* expressing hGrx1-roGFP2 and monitored for 9 min. Ratio images (405/488 nm) of the cells at different time points are provided (**A**, **C**). Furthermore, the ratios 405/488 nm were computed and plotted against time (**B**, **D**). For each concentration data from 3 trophozoites were analyzed. Standard errors of the mean were not greater than 15%.

**Figure 5:**
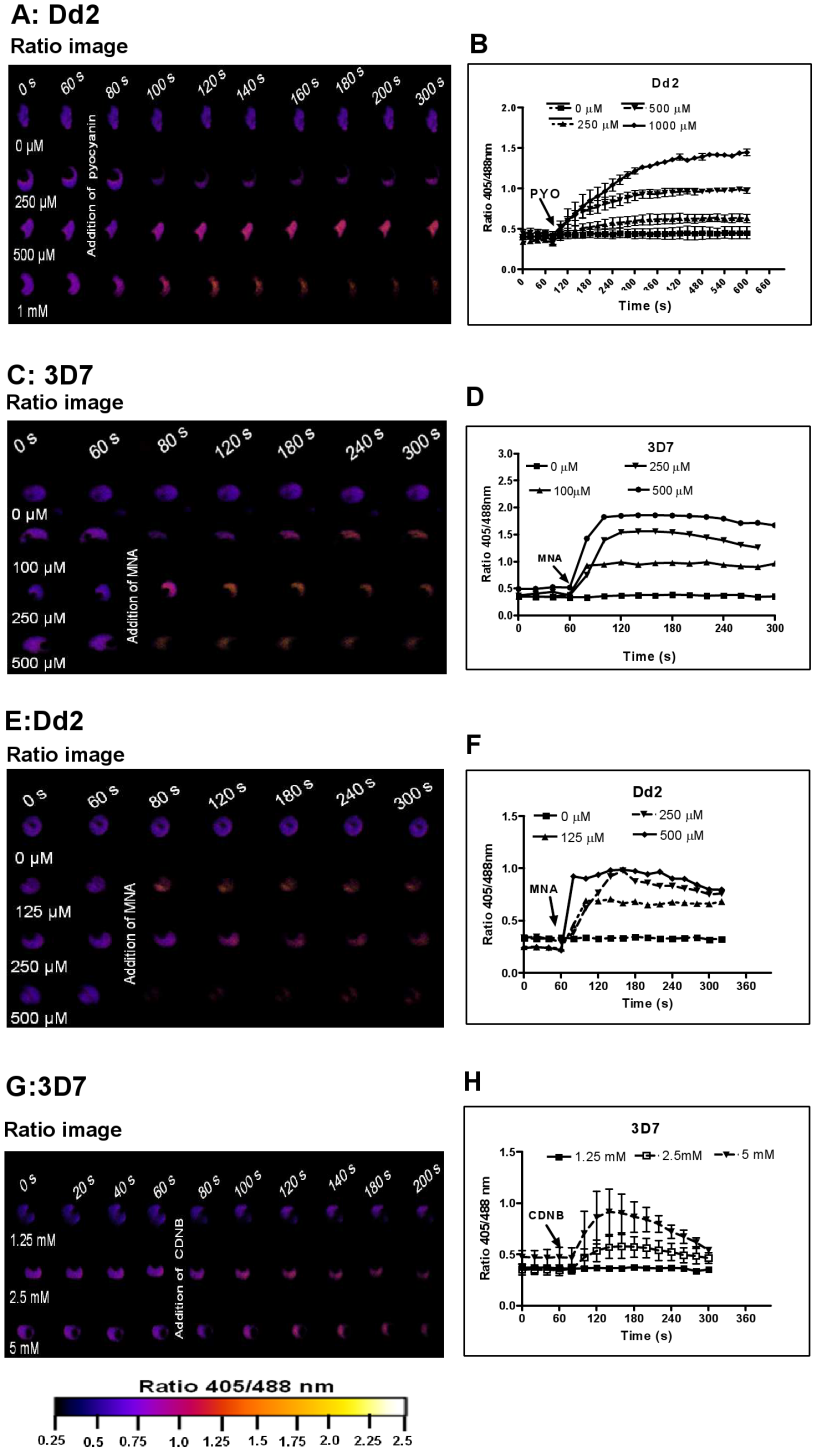
hGrx1-roGFP2 visualizes cytosolic depletion of glutathione induced by pyocyanin, menadione, and CDNB. *P. falciparum* parasites expressing hGrx1-roGFP2 were treated with micromolar concentrations of pyocyanin (PYO) (**A**, **B** Dd2**)**, menadione (MNA) (**C**, **D** 3D7; **E**, **F** Dd2), and 1-chloro-2,4-dinitrobenzene (CDNB: **G**, **H** 3D7). After 60 s preincubation, *P. falciparum* parasites expressing hGrx1-roGFP2 were treated with different concentrations of PYO, MNA, and CDNB and monitored for 4 min. False color ratio (405/488 nm) images at different time points are depicted. The ratio of emissions after excitation at 405 and 488 nm was computed and plotted against time. For each concentration, data from 3 trophozoites were analyzed. Mean and standard errors of the mean are shown.

Prior to investigating the effects of antimalarial drugs and redox-active compounds on hGrx1-roGFP2 in living cells, we (re)-assessed the IC_50_ values of the drugs in a standardized IC_50_ assay, which is based on a 72-hour incubation and incorporation of ^3^H-labeled hypoxanthine (please see details in Table 1 and the Materials and Methods section). Then, we first tested short-term incubations (up to 5 min) for which rather high drug concentrations were necessary in order to observe effects (for a summary please see Table 2). Among the antimalarial drugs tested, only MB evoked an immediate increase in the fluorescence ratio 405/488 nm; this increase was observed in both parasite strains (3D7: Fig. 4A, B; Dd2: Fig. 4C, D) and was faster and higher in 3D7 than in Dd2. A direct interaction between MB and the probe (see above) definitely has to be considered when interpreting this result. Interestingly, PYO, a natural compound structurally related to MB, (Figs. 5A, B) and MNA also caused an increase in the fluorescence ratio 405/488 nm (Figs. 5C, D) and Dd2 (Figs. 5E, F) within 5 min. Effects were also observed for 1-chloro-2,4-dinitrobenzene (CDNB), a thioredoxin reductase inhibitor and an electrophilic xenobiotic compound that is detoxified by conjugation to GSH [33]. On the 3D7 strain, millimolar concentrations of CDNB caused a pronounced but transient increase in the fluorescence ratio (Figs. 5G, H). In contrast (and as expected), 1 mM of the glutathione biosynthesis inhibitor L-buthionine sulfoximine (BSO) did not immediately induce ratio changes (shown for Dd2^hGrx1-roGFP2^ in Fig. S6A). Artemisinin (Fig. S6B–D) and quinoline-based (Fig. S7) antimalarials at 100 µM did not change the fluorescence signal of the probe within 5 min, either.

**Table 2:** Short-term effects of redox-active compounds and antimalarial drugs on the redox ratio of hGrx1-roGFP2 in living parasites.

Drugs/redox-active compounds (abbreviations)	*P. falciparum* 3D7 strain		*P. falciparum* Dd2 strain	
	Increase in fluorescence ratio	Fold change of fluorescence ratio	Increase in fluorescence ratio	Fold change of fluorescence ratio
Diamide (control) (DMD)	0.50→1.79 (1 mM, 10 sec)	3.58 (1 mM, 10 sec)	0.37→1.49 (1 mM, 10 sec)	4.03 (1 mM, 10 sec)
**ROS generators**				
Hydrogen peroxide (H_2_O_2_)	0.55→2.21 (20 mM, 20 sec)	4.02 (20 mM, 20 sec)	0.31→0.95 (20 mM, 20 sec	3.06 (20 mM, 20 sec)
*tert*-Butyl hydroperoxide (TBHP)	nd		0.38→1.05 (10 mM, 4 min)	2.76 (10 mM, 4 min)
Paraquat (PQT)	No major effect (1 mM, 1 min)		No major effect (1 mM, 1 min)	
**RNS donors**				
Sodium nitroprusside (SNP)	No major effect (1 mM, 1 min)		No major effect (1 mM, 1 min)	
3-Morpholino-sydnonimine hydrochloride (SIN1)	nd		0.38→1.10 (10 mM, 4 min)	2.89 (10 mM, 4 min)
**Redox cyclers, inhibitors of GSH synthesis, xenobiotics**				
Methylene blue (MB)	0.50→2.0 (50 µM, 1 min)	4.00 (50 µM, 1 min)	0.29→1.31 (50 µM, 5 min)	4.52 (50 µM, 5 min)
Pyocyanin (PYO)	No major effect (1 mM, 5 min)		0.38→1.33 (1 mM, 5 min)	3.5 (1 mM, 5 min)
Menadione (MNA)	0.50→1.80 (500 µM, 1 min)	3.60 (500 µM, 1 min)	0.35→0.91 (500 µM, 1 min)	2.60 (500 µM, 1 min)
L-Buthionine sulfoximine (BSO)	No major effect (1 mM, 5 min)		No major effect (1 mM, 5 min)	
1-chloro-2,4-dinitrobenzene (CDNB)	0.47→0.89 (5 mM, 90 sec)	1.89 (5 mM, 90 sec)	nd	
**Quinoline drugs**				
Chloroquine (CQ)	No major effect (100 µM, 5 min)		No major effect (100 µM, 5 min)	
Amodiaquine (AQ)	No major effect (100 µM, 5 min)		No major effect (100 µM, 5 min)	
Quinine (QN)	No major effect (100 µM, 5 min)		No major effect (100 µM, 5 min)	
Mefloquine (MQ)	No major effect (100 µM, 5 min)		No major effect (100 µM, 5 min)	
**Artemisinin derivatives**				
Artemisinin (ART)	No major effect (100 µM, 5 min)		No major effect (100 µM, 5 min)	
Artesunate (ATS)	No major effect (100 µM, 5 min)		No major effect (100 µM, 5 min)	
Artemether (ATM)	No major effect (100 µM, 5 min)		No major effect (100 µM, 5 min)	

Values are given for representative time points and drug concentrations of particular interest. For complete dose or time/response curves please refer to the figures.

### Effects of antimalarial drugs on the glutathione redox potential of *Plasmodium* after 4 h incubation

The clinically employed concentrations of antimalarials are usually in the nanomolar range and thus much lower than the 100 µM employed above to see maximal effects in short-term incubations. Therefore we investigated whether hGrx1-roGFP2 can monitor changes in *E*_GSH_ at pharmacologically meaningful drug concentrations.

We incubated trophozoite stages of 3D7^hGrx1-roGFP2^ and Dd2^hGrx1-roGFP2^ for 4 h at drug concentrations ranging from ∼1×IC_50_ to 100×IC_50_. Please see Table 1 for IC_50_ values; as an example, for chloroquine on 3D7 this would correspond to a range of 8.6 nM–860 nM.

**Figure 6:**
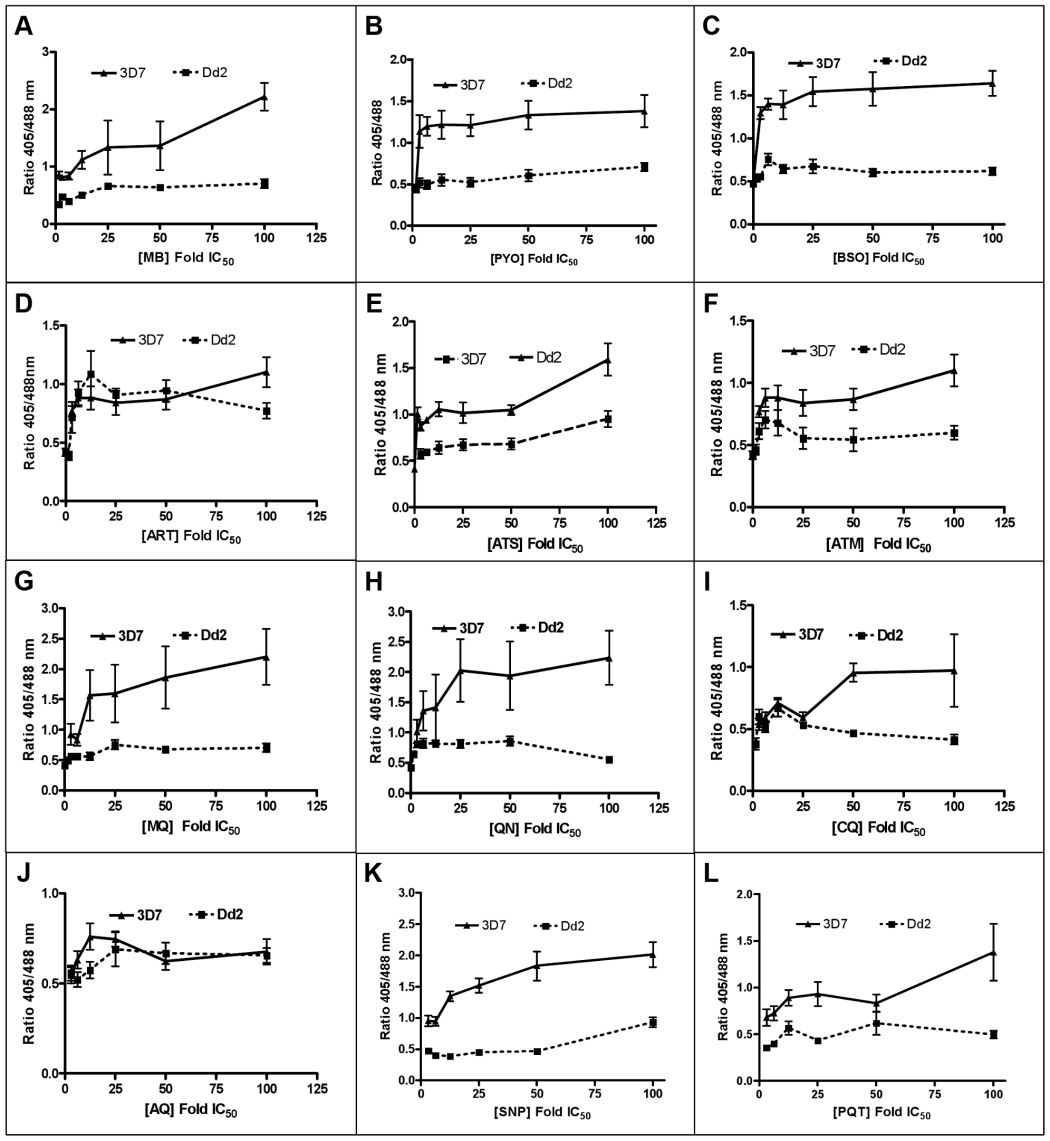
Effect of a 4*P. falciparum*. Trophozoite stage parasites (26–30 h) of the 3D7 and Dd2 strains of *P. falciparum* expressing hGrx1-roGFP2 were magnetically enriched (Miltenyi Biotec, Germany), then counted using the improved Neubauer hemocytometer (Brand GmbH, Germany), and returned to cell culture (at ∼5.0×10^3^ trophozoites/µl) for at least 2 h to recover. After the 2 h recovery period, the parasites were treated with the drugs at concentrations ranging from ∼1×IC_50_ to 100×IC_50_ for 4 h. Each drug concentration had 5 ml with ∼5.0×10^3^ trophozoites/µl. The parasites were excited with 405 and 488 nm lasers, and the ratio of emissions in the green channel (500–530 nm) was calculated. The ratio of emissions after excitation at 405 and 488 nm (ratio 405/488 nm) was plotted against the drug concentration. Results are shown for (**A**) methylene blue (MB), (**B**) pyocyanin (PYO), (**C**) buthionine sulfoximine (BSO), (**D**) artemisinin (ART), (**E**) artesunate (ATS), (**F**) artemether (ATM), (**G**) mefloquine (MQ), (**H**) quinine (QN), (**I**) chloroquine (CQ), (**J**) amodiaquine (AQ), (**K**) sodium nitroprusside (SNP), and (**L**) paraquat (PQT). Each data point comprises at least 8 trophozoites. Mean and standard errors of the mean are shown.

Following 4 h incubation with MB, PYO, and BSO, the fluorescence ratio 405/488 nm increased in both strains yet was more pronounced in 3D7 (Figs. 6A–C). Similar patterns were observed after 4 h treatment with ART, ATS, and ATM, although the ratio increase and differences between the two strains were less pronounced (Figs. 6D–F). MQ, QN, CQ, and AQ also induced dose-dependent changes in the fluorescence ratio, which were stronger in 3D7. Interestingly, for both strains much higher ratio changes were observed with MQ and QN than with CQ and AQ (Figs. 6G–J). Additionally, we evaluated the effects of SNP and PQT (Figs. 6K–L).

### Effects of antimalarial drugs on the glutathione redox potential of *Plasmodium* after 24 h incubation

**Figure 7:**
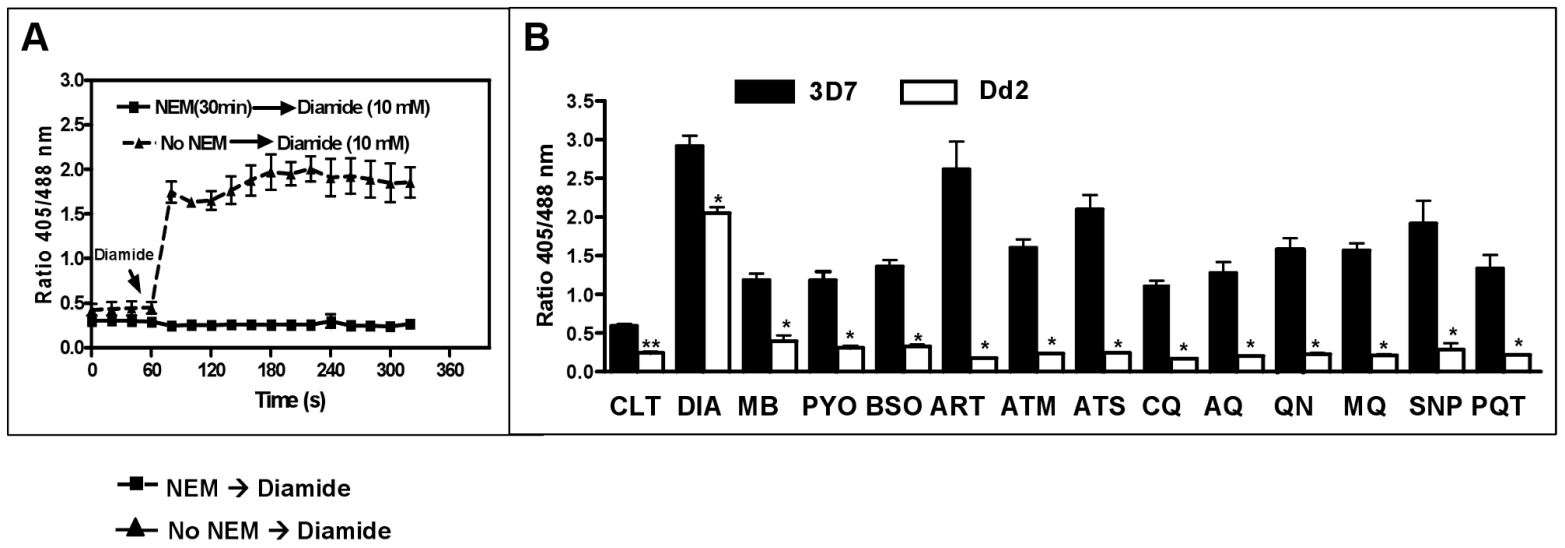
Changes in the glutathione redox potential in *P. falciparum* via 24 h incubation with antimalarial drugs. A ring stage culture (10 ml, 2.5% hematocrit, 3–4% parasitemia) of 3D7 (dark bars) or Dd2 (white bars) strains of *P. falciparum* expressing hGrx1-roGFP2 were treated with antimalarial drugs at a concentration of 4×IC_50_ for 24 h. (**A**) Preliminary experiments established that 20 mM NEM (30 min) induces instant clamping of the hGrx1-roGFP2 redox state, which could not be reverted by adding 10 mM diamide. (**B**) Following 24 h incubation, cultures were treated with 20 mM NEM for 30 min [32], enriched by magnetic separation (Miltenyi Biotec, Germany), and fluorescence was immediately measured. The parasites were excited with 405 and 488 nm laser wavelengths, and the ratio of emissions (405/488 nm) in the green channel (500–530 nm) was calculated. All experiments included a negative control (no drug treatment, CTL) and a positive control (treatment with 1 mM diamide (DIA)). Results are shown for methylene blue (MB), pyocyanin (PYO), buthionine sulfoximine (BSO), artemisinin (ART), artemether (ATM), artesunate (ATS), chloroquine (CQ), amodiaquine (AQ), quinine (QN), mefloquine (MQ), sodium nitroprusside (SNP), and paraquat (PQT). For each drug, the data (mean ± SEM) represent 10–15 (3D7) and 25–30 (Dd2) trophozoites. The fluorescence ratio 405/488 nm values for the 3D7 strain were significantly different from those of the Dd2 strain as indicated (**, p<0.001; *, p<0.0001).

MB [38], artemisinin derivatives [39], and quinoline drugs [15] exert differential stage-specific antimalarial activity. Accordingly, we investigated whether hGrx1-roGFP2 can be used to monitor the effect of antimalarial drugs on *E*_GSH_ during development from ring to trophozoite stages. We treated ring stages of 3D7^hGrx1-roGFP2^ and Dd2^hGrx1-roGFP2^ for 24 h with 4×IC_50_ concentrations of the different drugs, which were in the lower nanomolar range for most compounds. Before starting these experiments, we verified that 20 mM *N*-ethylmaleimide (NEM) led to an instant clamping of the cytosolic redox state determined by hGrx1-roGFP2, as previously reported [32] (Fig. 7A). This information was essential, since we had to clamp the current redox state before enriching the parasites after the 24 h incubation via magnetic separation and measuring the redox potential (see Methods).

One mM diamide served as a maximally oxidizing control for oxidation and resulted in a pronounced increase in redox potential in both strains; in some cells even cell lysis was observed. For all other compounds tested, a clear decrease of the fluorescence ratio was observed in the 3D7 strain, whereas the Dd2 strain seemed to be much less susceptible (Fig. 7B). Interestingly, artemisinin derivatives had the strongest effect on the redox potential.

### Parallel determination of redox parameters

In order to verify that indeed specific changes in the cellular glutathione redox milieu occur under the experimental conditions chosen for the hGrx1-roGFP2 measurements, we determined different redox parameters in parasite cell extracts. Concentrations of total (protein-bound and free) thiols, total glutathione, and the redox state of thioredoxin 1 were measured in *P. falciparum* 3D7 after incubation with different drugs. For this purpose we employed spectrophotometric methods – the Ellman assay and the DTNB-recycling assay – as well as western blots for thioredoxin detection.

**Figure 8:**
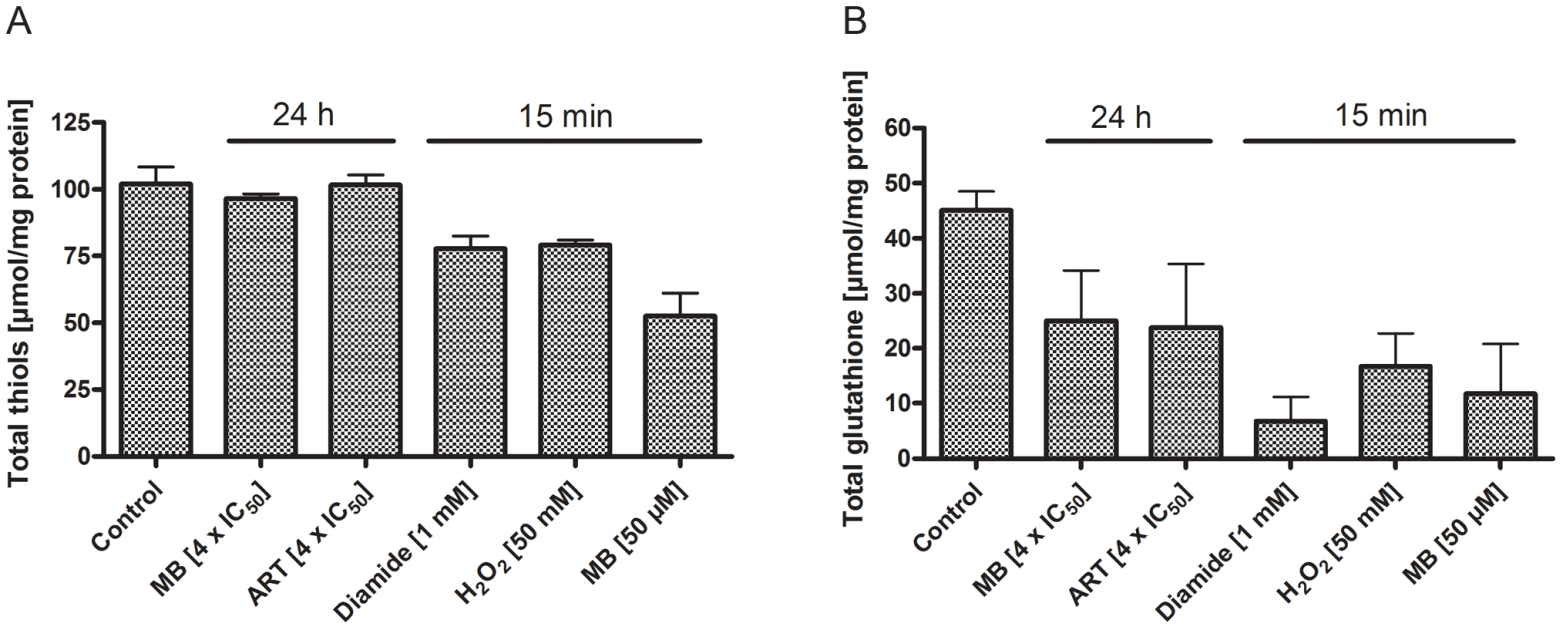
Total thiols and total glutathione concentrations in *P. falciparum* after drug treatment. Concentrations of total thiols (**A**) and total glutathione (**B**) in *P. falciparum* 3D7 after incubation with 4×IC_50_ of methylene blue (MB) or artemisinin (ART) for 24 h, or with 1 mM diamide, 50 mM H_2_O_2_ or 50 µM MB for 15 min. As indicated, the short-term incubations with high drug concentrations led to changes in the total thiol status, and a clear drop in glutathione concentrations was detectable under all conditions. After 15 min incubation with 1 mM diamide, cells started to lyse in most experiments; 24 h incubation with 1 mM diamide led to complete disruption of the parasites (data not shown).

As indicated in Fig. 8, the 15 min incubations with high drug concentrations (1 mM diamide, 50 mM H_2_O_2_, or 50 µM MB) led to changes in the total thiol status. All conditions – including the 24 h incubations at pharmacologically meaningful drug concentrations (4×IC_50_ of methylene blue or artemisinin) – led to a pronounced drop in glutathione concentrations.

Interestingly, only the most stressful condition (1 mM diamide), which led to partial loss of protein in short-term experiments and was only survived by a part of the cells at longer incubations, induced changes in the ratio of reduced to oxidized thioredoxin (Fig. S8).

## Discussion

### hGrx1-roGFP2 as a dynamic biosensor in *Plasmodium falciparum*

Due to the great importance of cellular redox reactions in malaria parasites and the mechanism of action of antimalarial drugs, it would be of great value to be able to follow the intracellular glutathione redox potential in living cells. We therefore tested the applicability of the ratiometric hGrx1-roGFP2 redox sensor in *Plasmodium falciparum*. The parasites could be transfected with the probe, and a stable expression of the protein without decomposition products was determined under (patho)physiologically and pharmacologically meaningful conditions. Furthermore, a disturbing influence of host cell hemoglobin autofluorescence could be ruled out.

In accordance with previous studies [31], we observed a rapid, dynamic, and ratiometric response of hGrx1-roGFP2 upon oxidation with diamide and reduction with DTT, which indicates its applicability as a live cell *E*_GSH_ biosensor in *P. falciparum*.

Interestingly, when comparing the basal hGrx1-roGFP2 redox ratios of the CQ-sensitive *P. falciparum* 3D7 strain and the CQ-resistant Dd2 strain, the latter constantly showed slightly lower values (0.59±0.09 in 3D7 vs. 0.29±0.08 in Dd2). This observation might at least partially be explained by higher concentrations of total glutathione in Dd2, which have been previously reported [21] and might induce a stronger basal reduction of hGrx1-roGFP2 in Dd2. The basal redox ratio of the hGrx1-roGFP2 sensor primarily depends on the GSH/GSSG ratio. However, as shown by Meyer *et al.* [33], the total glutathione concentration can also influence the fluorescence ratio of the sensor. This is not unexpected since the glutathione redox potential also depends on both the GSH/GSSG ratio and the glutathione concentration. Therefore, when employing the hGrx1-roGFP sensor to monitor drug effects in living *Plasmodium falciparum* parasites, it is important to thoroughly monitor the basal redox ratio and then follow the changes in direct comparison with control cells. When interpreting the results, one must keep in mind that the observed changes can be due to a change in the GSH/GSSG ratio and/or to changes in the total glutathione concentration [24], [33], which can occur, e.g., after GSSG export. Indeed, glutathione homeostasis has been shown to be differentially regulated in CQ- resistant and sensitive *P. falciparum* strains. This differential regulation seems to be mainly effected by glutathione biosynthesis, by PfGR, and by GSSG efflux [40], [21]. If the difference observed between our two strains is specific for the parasites used or represents a general difference between drug-sensitive and drug-resistant parasites, this needs to be addressed in further studies. So far different concentrations (0.4 mM to 2.3 mM) of total cytosolic glutathione have been reported in *P. falciparum* [19], [27], [41]. These discrepancies may be due to real strain-specific differences and/or to the use of cell-disruptive methods, which can result in a loss of glutathione, in mixing of glutathione from different compartments, in oxidative processes (e.g. enhancing glutathionylation reactions [42]), and in red blood cell contaminations. Many of these problems can be overcome by using the hGrx1-roGFP2 sensor.

Notably, when estimating the basal *E*_GSH_ in the cytosol on the basis of the fluorescence after maximal oxidation (1 mM diamide) and after full reduction (10 mM DTT) [31], [33], we obtained values of −314.2±3.1 mV and −313.9±3.4 mV for 3D7 and Dd2, respectively. These values did not differ significantly and are also supported by previous studies [43] using a pH-sensitive green fluorescent protein (pHluorin), in which no significant differences in the cytosolic pH were found between the CQ-sensitive HB3 (pH = 7.03±0.09) and CQ-resistant Dd2 strain (pH = 7.20±0.06). Other studies using different methodological approaches reported pH values of 7.29±0.01 for the FAF-6 strain [44] and 7.31±0.02 for the FCR-3 strain [45]. Using the hGrx1-roGFP2 sensor systematically in parallel with other methodological approaches, it is now possible to approach the direct comparison of basal redox ratios, redox potentials, and pH values in various *Plasmodium* strains.

The basal *E*_GSH_ of Dd2 and 3D7 indicate that the parasites' cytosol is more reducing than suggested in previous estimates [22]. Furthermore, our data are comparable to the cytosolic *E*_GSH_ determined by roGFP in other organisms such as −325 mV in HeLa cells [31], −318 mV in *Arabidopsis thaliana* [33], and −310 to −320 mV in *Saccharomyces cerevisiae* [28], [46].

The dynamic range of hGrx1-roGFP2 was determined to be 6.36±0.73 and 5.28±0.49 in 3D7 and Dd2, respectively. These values are also in agreement with previously reported dynamic ranges of hGrx1-roGFP2 in other organisms of 4.4 [32] and 4 to 8 [28]. The data suggest furthermore that the presence of higher GSH levels confers a greater redox buffering capacity in the Dd2 strain compared to the 3D7 strain.

Treatment of cells with H_2_O_2_ is a common tool for probing the sensitivity of biosensors to oxidative stress [28]. In 3D7 and Dd2, H_2_O_2_ also led to a rapid increase in the fluorescence ratio of the probe; however, millimolar concentrations of H_2_O_2_ were required to see this effect. As reported in [32] and shown in Table 1, the hGrx1-roGFP2 biosensor is not sensitive to direct interaction with micromolar H_2_O_2_ concentrations. However, millimolar H_2_O_2_ might lead to a direct oxidation of the probe. Since, however, H_2_O_2_ is likely to be at least partially detoxified by the antioxidative defense systems of the parasite-host cell unit, an oxidizing effect on the glutathione system also needs to be considered. The long and steady increase in the ratio after treatment with THBP might be associated to the fact that TBHP is, in contrast to H_2_O_2_, not detoxified by red blood cell catalase and is therefore likely to have more pronounced and longer-lasting effects.

Induction of nitrosative stress using the peroxynitrite generator SIN-1 also led to a rapid ratio change. Therefore, we assume that ROS and RNS do affect *E*_GSH_ in *P. falciparum* and that hGrx1-roGFP2 seems to be a suitable tool to monitor these changes in living cells.

However, when using the redox probe, one has to take into account that some compounds, independent from glutathione, might also directly interact with the probe. Thus, before testing the effects of antimalarial compounds on the ratio of the probe in living parasites, we characterized their direct *in vitro* interaction with recombinant hGrx1-roGFP2. Table 1 and Figs. 3 and S5 summarize the data for different concentrations and incubation times. Whereas high concentrations of oxidizing agents and redox cyclers such as GSSG, diamide, H_2_O_2_, MB, and PYO led to direct interactions with the probe, antimalarial drugs including the quinolines and artemisinin derivatives did not cause a major increase in the fluorescence ratio even at concentrations up to 100 µM and after 24 h incubation. These data can of course only be indirectly compared to the situation *in vivo*, where a direct interaction is hindered by multiple cell membranes, degradation of the stressors, and binding to other proteins. However, the data are important when interpreting the results described in the next paragraph. Whereas compounds such as MB might induce changes in the probe via direct interaction and by acting on the cellular redox metabolism, the direct interactions of the other drugs with the probe seem to be negligible. Furthermore, effects induced by diamide or H_2_O_2_ might recover more rapidly than those induced by potent redox cyclers such as MB and pyocyanin.

### Effects of antimalarial drugs on the cytosolic glutathione redox potential

In order to investigate whether hGrx1-roGFP2 can be used to monitor changes in *E*_GSH_ after the treatment of cell cultures with antimalarial drugs, we incubated the *Plasmodium falciparum* strains 3D7 and Dd2 with different concentrations of MB, quinoline, and artemisinin derivatives in short-term (5 min), medium-term (4 h), and long-term (24 h) experiments (Table 2, Figs. 4–7). Interestingly, in almost all experiments the observed ratio changes were stronger and faster in the 3D7 strain when compared to the Dd2 strain. As discussed above, this might be due to higher absolute GSH levels in the Dd2 strain that confer a greater redox buffering capacity. This hypothesis is supported by the lower basal fluorescence ratio and the lower dynamic range observed in Dd2.

In the short-term experiments, MB rapidly evoked hGrx1-roGFP2 ratio changes. This might partially be due to a direct interaction with the probe; however, as indicated in Fig. 8, 50 µM MB also induced a marked decrease in cellular total thiol and glutathione concentrations within minutes. As for most other conditions, the increase in ratio was faster and higher in 3D7 than in Dd2. The following relatively fast decline in the ratio in 3D7 might be explained by the fact that the initial increase was much higher (about 2.0 in 3D7 and only about 1.3 in Dd2 at 50 µM). Additionally, 24 h incubations with 4×IC_50_ MB, corresponding to only 13.2 nM in 3D7, led to strong changes in the glutathione redox potential and to glutathione depletion. At these concentrations a direct interaction with the probe is very unlikely. MB is the oldest synthetic antimalarial drug [47] and has been shown to be active against asexual parasite stages [38] and against gametocytes both *in vitro* [36] and in clinical studies [48]. Due to reduced susceptibility of *P. falciparum* strains to artemisinin derivatives, there has recently been a renewed interest in MB-based combination therapies [49], [50].

As expected, BSO, an inhibitor of glutathione biosynthesis, also increased the redox ratio of the probe within 4 h. It is well known that BSO leads to GSH depletion within a relatively short period of time – not only in parasites but also other cells that depend on GSH biosynthesis [21]. Therefore, the changes observed in the redox sensor are most likely due to diminished glutathione levels rather than to a shift in the GSH/GSSG ratio. This might also explain why the changes observed were so much stronger in 3D7 (which has relatively low basal glutathione levels) than in Dd2.

Artemisinin-based (Fig. S6 B–D) antimalarials at 100 µM did not rapidly (within 5 min) oxidize the parasites' cytosol. This result is consistent with the fact that artemisinin derivatives produce carbon-centered free radicals reacting with GSH [9], [10] and are not cyclically oxidized or reduced as MB and PYO are. Therefore, only one free radical can result from one drug molecule. However, like quinolines [11], artemisinin-based [6] antimalarials have been shown to accumulate in the food vacuole and inhibit heme polymerization, resulting in pro-oxidative byproducts. In accordance with this, artemisinins led to pronounced effects on the cellular glutathione redox potential after 4 h and 24 h incubation. The data are substantiated by a decrease of glutathione concentrations as determined in our 24 h experiments (Fig. 8). ART and its derivatives ATS and ATM are the mainstay of antimalarial treatment as major partners in combination therapy (ACT) [2]. Artemisinin-based derivatives are characterized by a broad activity across asexual stages including ring, trophozoite and schizont stages as well as against young gametocytes [2]. In *P. falciparum*, using a fluorescent dansyl trioxane derivative [51] and the pH-sensitive probe LysoSensor Blue [52], ART has been shown to initially accumulate in the food vacuole. Pro-oxidant end products have, however, only been observed at high concentrations (>100 µM) [9], whereas the drug is effective at 10,000-fold lower concentrations [38]. In our 24 h incubation at 4×IC_50_, the effects of artemisinin derivatives were stronger in 3D7 than in Dd2 parasites, although the susceptibility of the strains to ART and its derivatives is comparable Table 1, [38]. Taken together, our data indicate that ART and its derivatives exhibit significant but delayed effects on glutathione metabolism. One could hypothesize that these endoperoxide drugs accumulate and act on targets that lead to the production of reactive oxygen species, which ultimately affect cytosolic GSH levels. Putative targets of alkylation such as the PfATP6 – a SERCA-type Ca^2+^ – ATPase [53], the translationally controlled tumor protein (TCTP) [54], heme [55], reduced GSH [56], and proteins of the electron transport chain [57] have been reported.

Quinoline drugs at 100 µM also did not induce major short-term effects on the *E*_GSH_ (Fig. S7); however, they dose-dependently oxidized the cytosol after 4 hours (Fig. 6). After 24 h at 4×IC_50_ pronounced effects were also observed (Fig. 7). Interestingly, a stronger oxidation was observed with MQ and QN than with CQ and AQ. Before adopting ACT, quinoline antimalarial drugs including CQ, AQ, QN, and MQ played a key role in antimalarial chemotherapy [2]. Due to high resistance development, the use of CQ was reduced, but AQ and MQ continued to play major roles as partner drugs in ACT. Furthermore, QN is recommended for severe malaria [58]. Notably, mutations mainly in *pfcrt* [59] and *pfmdr1* [60] differentially influence quinoline drug action and resistance. However, an additional role for GSH in its mode of action and resistance to CQ was also suggested [14]; quinoline antimalarials can inhibit the biocrystallization of FP into hemozoin in the food vacuole. Free FP may exit from the food vacuole into the parasite's cytosol where it can be degraded with GSH as a cofactor. GSH-dependent FP degradation is reported to be effectively inhibited by CQ and AQ but not by QN or MQ [14]. Furthermore, a possible role for CQ-FP-induced oxidative stress as part of the mechanism of action of CQ has been proposed [61]; however, this hypothesis has been questioned by others [16].

Since even 100 µM of the quinoline and artemisinin drugs hardly influenced the fluorescence ratio of isolated recombinant hGrx1-roGFP2 over 24 h, the redox effects on living cells are likely to represent real drug-induced changes. Interestingly, none of the drug incubations tested led to oxidation of the redox protein thioredoxin 1. Only the most stressful and artificial condition (24 h, 1 mM diamide), which led to cell disruption and loss of protein, induced (hardly detectable) changes in the ratio of reduced to oxidized thioredoxin (Fig. S8). These data indicate that – in comparison with other systems [62] – *Plasmodium falciparum* thioredoxin 1 seems to be particularly robust towards oxidative stress. The data further indicate that the hGrx1-roGFP2 sensor is likely to specifically determine changes in the parasites' glutathione system.

### Conclusion

Our data indicate that hGrx1-roGFP2 is a sensitive and reliable biosensor for assessing and quantifying real-time changes in the cytosolic glutathione redox potential of the malaria parasite *Plasmodium falciparum*. The sensor allows live cell spatiotemporal resolution of the parasite's GSH metabolism for the first time. This tool will be most valuable not only for further understanding physiologic adaptations of the cellular redox potential but also for studying redox-related mechanisms of drug action and resistance and for comparing different redox-related methodological approaches. Depletion of GSH may cause disruption of vital cellular processes in *P. falciparum* and is a possible but not necessary sign of incipient parasite death [63]. Oxidative and nitrosative stress are furthermore known to promote protein *S*-glutathionylation [64], nitrosylation, and sulfenylation, which can regulate and affect central redox-regulated metabolic processes. Recently, 493 targets of protein *S*-glutathionylation were identified in *P. falciparum* [42]. To further understand redox regulatory processes in *Plasmodium*, mechanisms of drug action, and the effects of host-derived oxidative immune response, the hGrx1-roGFP-based biosensor will be an invaluable tool. Furthermore, attempts to specifically target the sensor to other subcellular compartments, including mitochondria and apicoplast, are currently underway in our laboratory.

## Materials and Methods

### Drugs and chemicals

All chemicals used were of the highest available purity and were obtained from Roth (Karlsruhe, Germany), Sigma-Aldrich (Steinheim, Germany), or Merck (Darmstadt, Germany). RPMI 1640 medium was from Gibco (Paisley, United Kingdom). Methylene blue was obtained from Roth (Karlsruhe, Germany); CQ and amodiaquine were from Sigma-Aldrich; mefloquine was from Roche (Mannheim, Germany); artemisinin was from Aldrich Chemical Co. (Milwaukee, Wis.); quinine was from Acros Organics (Geel, Belgium); and pyocyanin was from Cayman Chemical (Ann Arbor, MI). Artemisinin derivatives (artemether and artesunate) were kindly provided by the Swiss Tropical Institute (Basel, Switzerland). WR99210 was kindly supplied by D. Jacobus, Princeton, New Jersey, USA. Anti-GFP antibody was from Roche, Mannheim, Germany, and anti-Grx antibody was from Santa Cruz Biotechnology, Santa Cruz, CA, USA.

### *P. falciparum* cell culture

The 3D7 and Dd2 strains of *P. falciparum* were cultured as described [65]. The strains were propagated in RBC (A+) in complete medium (RPMI 1640 medium supplemented with 0.5% Albumax, 9 mM glucose, 0.2 mM hypoxanthine, 2.1 mM L-glutamine, and 22 µg/ml gentamycin) at 3.3% hematocrit and 37°C in a gaseous mixture consisting of 3% O_2_, 3% CO_2_, and 94% N_2_. Synchronization of *P. falciparum* parasites was carried out with 5% (w/v) sorbitol [66].

### *In vitro P. falciparum* drug susceptibility assays

To investigate the effect of oxidants and antimalarial drugs on *E*_GSH_, their activity against 3D7 and Dd2 strains of *P. falciparum* was first determined using the [^3^H] hypoxanthine incorporation assay [67]. Briefly, stock solutions of MB, CQ, BSO, PQT, and SNP were dissolved in sterile ddH_2_O, while AQ, QN, MQ, ART, ATM, and ATS were dissolved in DMSO. Dilutions were prepared in hypoxanthine-free medium. Serial double dilutions (100 µl) of the compounds were carried out in 96-well microtitre plates. Synchronized ring-stage parasites in hypoxanthine free complete medium (100 µl) were added to each well to a final volume of 200 µl (0.5% parasitemia and 2% hematocrit). After the 48 h incubation period at 37°C in a gaseous mixture (3% O_2_, 3% CO_2_, and 94% N_2_), 50 µl (final concentration of 0.5 µCi/well) of [^3^H]-hypoxanthine was added per well, and the plate was further incubated for 24 h. Following incubation (total of 72 h), the plates were frozen at −80°C for at least 1 h. Plates were then thawed; each well was harvested on a glass fiber filter (Perkin-Elmer, Rodgau-Jügesheim, Germany) and dried; and radioactivity in counts per minute (cpm) from each well was measured. The IC_50_s were determined by curve-fitting the percentage of growth inhibition (in relation to controls) against the log of drug concentration with a variable slope sigmoidal function (Prism 4.0 GraphPad Software, San Diego, CA). The resulting IC_50_ values summarized in Table 1 were used to orient our experiments and give the reader an impression of the pharmacological range in which the experiments took place. In order to observe short-term redox effects for most antimalarial drugs, much higher concentrations had to be applied (e.g. 100×IC_50_, which is still in the nanomolar to low micromolar range). Even at these rather high concentrations, the cells were still fully alive since the incubation time was only 4 h. In the 24 h experiments, a concentration of 4×IC_50_ was used since this was the dose at which moderate effects on the redox milieu were seen. As indicated by pretests based on light microscopy, growth, and multiplication rate of the parasites (see e.g. [68]) as well as indicated by our live cell imaging, >50% of the cells were still viable at 4×IC_50_ after 24 h (50% were still viable after 72 h as indicated by the IC_50_). However, it is likely that the effects on the redox milieu that we observed using the hGrx1-roGFP2 sensor represent a mixture of direct drug effects and indirect effects observed in stressed cells.

### Cloning the hGrx1-roGFP2 constructs

The hGrx1-roGFP2 coding sequence [69] was amplified using the forward (Grx1 primer: 5′ - AGTCGGTACCATGGCTCAAGAGTTTGTGAACT - 3′) and reverse (roGFP2 primer: 5′- AACCCCCGGGTTACTTGTACAGCTCGTCCATG -3′) primers and cloned into the pARL+ expression vector [34], using *Kpn*I and *Xma*I restriction sites (underlined).

### Transfection of *P. falciparum*

Transfection of *P. falciparum* was carried out as described [34]. Briefly, a 5 ml culture (ring stage 8–10 h, 5–8% parasitemia, 4% hematocrit) was centrifuged (1,500×g, 5 min), and the supernatant was aspirated. The parasite pellet (200 µl) was mixed with 150 µg of purified plasmid (pARL-1a+ hGrx1-roGFP2) in 400 µl of cytomix and then electroporated at 0.310 kV and 950 µF (Gene pulser, Bio-Rad) as described [34]. The resulting time constant was between 7 and 12 sec. The electroporated sample was returned to a 10 ml culture with 3.3% final hematocrit. To select for transfectants, 6 h post transfection, 2 nM WR99210 was added to the culture and later increased, usually after 3–4 weeks (after the appearance of transfectants), to 5 nM. The complete RPMI medium (with 2 nM or 5 nM of WR99210) was changed every day, and 100 µl of fresh RBCs were added every week.

### Western blot of the hGrx1-roGFP2 probe

Transfected *P. falciparum 3D7* trophozoite stage parasites (30–34 h) were harvested by saponine lysis as described in [70]. Parasite cultures were centrifuged, the pellets resuspended with 20 volumes of saponin lysis buffer (0.02% saponin, 10 mM NaH_2_PO_4_, 10 mM Na_2_HPO_4_, 145 mM NaCl, 3 mM KCL, pH 7.2), incubated for 10 min at 37°C, and washed three times with phosphate-buffered saline. For preparing the parasite cell extract, pellets were diluted in equal volumes of phosphate-buffered saline, and a complete protease inhibitor cocktail (Roche, Mannheim, Germany) was added. Parasites were disrupted by four cycles of freezing in liquid nitrogen and thawing in a water bath at room temperature. After centrifugation at 100,000 g for 30 min at 4°C, the supernatant obtained was used for western blotting. 20 µg of proteins from the parasite lysate were separated onto 16% SDS gels and transferred to a PVDF membrane (Roth, Karlsruhe, Germany). Membranes were probed with anti-GFP (1∶1,000; Roche) and anti-Grx (1∶5,000; Santa Cruz Biotechnology) and followed by secondary anti-mouse (1∶2,000; dianova, Hamburg, Germany) and anti-rabbit (1∶10,000, dianova, Hamburg, Germany) antibodies, respectively. All antibodies for western blotting were diluted in 5% non-fat milk in TBST.

### Thioredoxin-redox western blots

Stock solutions of MB were dissolved in distilled H_2_O, diamide in RPMI medium, and ART in DMSO. *P. falciparum* 3D7-infected erythrocytes (ring stage 6–10 h post invasion) were incubated with 4×IC_50_ of MB; 4×IC_50_ of ART; or 1 mM, 500 µM, 100 µM, 50 µM, or 10 µM of diamide for 24 h. For 15 min drug treatment, trophozoites (30–34 h post invasion) were incubated with 50 µM MB, 1 mM diamide, or 50 mM H_2_O_2_. 50 mM iodoacetic acid was added to prevent the formation of disulfide bonds of reduced cysteine residues, and samples were stored at −80°C.

Recombinant thioredoxin was reduced by 5 mM DTT for 30 min, desalinated via a micro bio-spin column with Bio-Spin p-6 desalinating columns (Biorad, Munich, Germany), and re-formation of disulfide bonds was prevented by treating it with 10 mM iodoacetic acid.

Cell lysate was obtained as described above. Samples were subjected to a native 16% PAGE and the reduced, partially oxidized, and fully oxidized Trxs were separated by electrophoresis [62]. The proteins were electroblotted onto a PVDF membrane (Roth, Karlsruhe, Germany). Membranes were probed with anti-PfTrx1 antibody (1∶5,000) followed by anti-rabbit antibody (1∶10,000, dianova, Hamburg, Germany). All antibodies for western blotting were diluted in 5% non-fat milk in TBST.

### Effect of antimalarial drugs on redox homeostasis in *P. falciparum*

To determine the effect of antimalarial drugs and classic redox agents on the *E*_GSH_ in *P. falciparum*, short-term (time series/course for 5 or 10 min), 4 h, and 24 h incubation experiments were carried out. For the short-term and 4 h experiments, trophozoite stage parasites (26–30 h) of the 3D7 and Dd2 strains expressing hGrx1-roGFP2 were magnetically enriched initially (Miltenyi Biotec, Germany) [62], [71] then counted using the improved Neubauer hemocytometer (Brand GmbH, Germany) and returned to cell culture (at ∼5.0×10^3^ trophozoites/µl) for at least 2 h to recover. For the short-term experiments, the classical oxidants diamide, H_2_O_2_, 3-morpholinosydnonimine hydrochloride, the reducing agent DTT, and antimalarial drugs (Table S1) were added to trophozoite stage parasites (26–30 h post invasion) of the 3D7 and Dd2 strains expressing hGrx1-roGFP2 and monitored for 4 or 9 min after 1 min of basal measurements with imaging every 10 or 20 s, respectively. For 4 h incubation experiments, trophozoite stage parasites (26–30 h) expressing hGrx1-roGFP2 were treated with antimalarial drugs at ∼1×IC_50_ to 100×IC_50_ for 4 h. Each drug concentration was added to 5 ml cell culture with ∼5.0×10^3^ trophozoites/µl. For 24 h experiments, a 10 ml culture (2.5% hematocrit, 3–4% parasitemia) of ring stage 3D7 or Dd2 strains expressing hGrx1-roGFP2 were treated with antimalarial drugs at 4×IC_50_ for 24 h. Following incubation, cultures were treated with 20 mM NEM for 30 min [32] and then enriched by magnetic separation (Miltenyi Biotec, Germany). Additionally, all experiments included a negative control (no drug treatment) and a positive control (treated with 1 mM diamide), and all measurements were done immediately after incubation. All experiments were carried out within 3–4 weeks after the appearance of transfectants.

### Determination of total thiol and total glutathione concentrations

Stock solutions of MB and diamide were dissolved in distilled H_2_O and ART in DMSO. *P. falciparum* 3D7-infected erythrocytes (ring stage 6–10 h post invasion) were incubated with 4×IC_50_ of MB, 4×IC_50_ of ART or 1 mM diamide for 24 h. For 15 min drug treatment, trophozoites (30–34 h post invasion) were incubated with 50 µM MB, 1 mM diamide or 50 mM H_2_O_2_. Cell lysate was obtained as described above. The content of total thiols in the fresh parasite lysate was measured spectrophotometrically on the basis of their reaction with DTNB [72]. The lysate was mixed with 150 mM potassium phosphate buffer, pH 8.0 and 400 µM DTNB, and directly measured at 412 nm at room temperature. For determining total glutathione, parasite lysate was mixed with 3 volumes of 5% sulfosalicylic acid (SSA), centrifuged, and the supernatant was directly used. The glutathione concentration was measured by the GR-coupled 5,5′-dithiobis-(2-nitrobenzoic acid) (DTNB)-GSH-recycling assay [73]. The SSA supernatant was mixed with buffer containing 142 mM NaH_2_PO_4_, 6 mM EDTA, and 0.3 mg/ml NADPH, pH 7.5. After adding 0.6 mM DTNB, the mixture was incubated for 10 min at room temperature. Human GR (0.5 U/ml) was added, and DTNB increase was immediately detected for 30 sec. The graphs were plotted using the GraphPad Prism 4 software (San Diego CA USA). Every treatment group was prepared in four biological replicates; two replicates were pooled and measured in two technical replicates each, resulting in four data values for statistical analysis.

### Confocal live cell imaging and image processing

*P. falciparum*-infected erythrocytes (trophozoite stage 26–30 h post invasion) were washed three times with pre-warmed (37°C) Ringer's solution (122.5 mM NaCl, 5.4 mM KCl, 1.2 mM CaCl_2_, 0.8 mM MgCl_2_, 11 mM D-glucose, 25 mM Hepes, 1 mM NaH_2_PO_4_, pH 7.4) and seeded in 50 µl Ringer's solution on poly-L-lysin-coated μ-slides VI (Ibidi, Martinsried, Germany). Slides were kept at 37°C in a gaseous mixture consisting of 3% O_2_, 3% CO_2_, and 94% N_2_ for up to 30 min and then immediately used for microscopy not longer than 30 (usually only 5) min. The redox ratio of untreated cells under these conditions was determined to be stable for up to one hour. Drugs were freshly dissolved in pre-warmed (37°C) Ringer's solution. We used a Leica confocal system TCS SP5 inverted microscope equipped with the objective (HCX PL APO 63.0×1.30 GLYC 37°C UV) and a 37°C temperature chamber. The argon laser power was set to 20%. Scanning was performed at 400 Hz frequency. The smart gain and smart offset were 950 V and −0.9%, respectively. The microscope was calibrated with both fully oxidized and fully reduced *P. falciparum* strains expressing hGrx1-roGFP2. Via a sequential scan, we excited hGrx1-roGFP2 at 405 nm and at 488 nm and detected emissions in the green channel (500–530 nm). Laser intensity for both lines was adjusted to match the full dynamic range of the probe to the dynamic range of the detector. For time series, images were acquired every 10 s or 20 s at a 512×512 pixel resolution. We used Image J (http://rsbweb.nih.gov/ij/) and the Leica software to analyze the data. The region of interest used for analysis was always a well-defined cytosol compartment excluding vacuolar regions. Only parasites with intact food vacuoles were analyzed. Images were exported to the Image J software, and the background was subtracted. Subsequently, the ratio (405/488 nm) was computed by dividing the 405 nm by the 488 nm image pixel by pixel as described [32]. We used the Image J look-up table ‘Fire’ for creating false color ratio images. The basal *E*_GSH_ was calculated from fluorescence intensity measurements as described [31], [33]. The graphs were plotted using the GraphPad Prism 4 software (San Diego CA USA).

### Heterologous overexpression of recombinant hGrx1-roGFP2

For control experiments evaluating the interaction of isolated hGrx1-roGFP2 and the redox compounds tested, we expressed the recombinant hGrx1-roGFP2 protein as described in [74] with modifications. Briefly, the *E. coli* M15 strain (Qiagen) was transformed with pQE-60/hGrx1-roGFP2 plasmid. Next, a pre-culture of LB medium (3 ml containing 100 µg/ml carbenicillin and 50 µg/ml kanamycin) was inoculated with a colony and grown for 8 h at 37°C with vigorous shaking. Then 100 ml of 2YT medium (containing 100 µg/ml carbenicillin and 50 µg/ml kanamycin) were inoculated with 3 ml culture and grown at 37°C overnight. The overnight culture (∼20–30 ml) was added to 1,000 ml of 2YT medium (containing 100 µg/ml carbenicillin and 50 µg/ml kanamycin) up to an optical density at 600 nm (OD_600_) = 0.1 and grown at 37°C until OD_600_ = 0.6 before induction with 1 mM isopropyl-β-D-1- thiogalactopyranoside. Following induction, the culture was grown overnight at 25°C, and the cells were harvested via centrifugation (8,000 g for 15 min at 4°C). Then the pellet was re-suspended (1 g pellet/4 ml buffer) in 50 mM sodium phosphate buffer, 300 mM NaCl, pH 8.0, and mixed with protease inhibitors before storage at −20°C. The hGrx1-roGFP2 protein was purified via hexahistidyl affinity chromatography on Ni-NTA-material, concentrated and desalinated (Centri-Spin 20 columns, Princeton Separations Inc.), and stored at −80°C.

### *In vitro* interaction of antimalarial drugs with the hGrx1-roGFP2 protein

Stock solutions of CQ, MB, BSO, PQT, GSSG, diamide, SIN1, and SNP were dissolved in distilled H_2_O while AQ, QN, MQ, ART, ATS, and ATM were dissolved in DSMO. MNA and PYO were dissolved in methanol and ethanol, respectively. TBHP, H_2_O_2_, and all drugs were diluted with a standard reaction buffer (100 mM potassium phosphate, 1 mM EDTA, pH 7.0, N_2_ saturated) and used immediately. Initially, the reaction buffer (100 ml) was degassed for 1 h and then saturated with N_2_ for 2 h on ice. To calibrate the assay, all experiments included 1 mM diamide (or 10 mM H_2_O_2_) and 20 mM DTT as controls in order to achieve maximum oxidation and reduction, respectively. Purified hGrx1-roGFP2 protein was reduced with 20 mM DTT for 45 min on ice, desalinated (Zeba Desalt spin columns, Pierce), and diluted in reaction buffer to a final concentration of 1.25 µM. Then a 5 fold drug dilution (50 µl) was mixed with 200 µl of 1.25 µM hGrx1-roGFP2 in a 96-well plate (black, μClear TC Greiner). We measured the emission of hGrx1-roGFP2 (505–515 nm) after excitation at 390 and 480 nm in a plate reader (M200, Tecan) with optimal read setting. We calculated the ratio of the emission (405/488 nm) and plotted it against time or concentration of antimalarial drugs. An excitation spectrum was scanned from 340–512 nm with emission at 530–540 nm.

## Supporting Information

Figure S1 **The redox sensor Grx-roGFP in** ***P. falciparum*** **remains intact after drug treatment.** Western blots of Grx-roGFP transfected *P. falciparum* 3D7 with (**A**) anti-GFP and (**B**) anti-Grx antibody after incubation with different concentrations of methylene blue (MB), artemisinin (ART), or diamide for 4 or 24 h. Incubation with 1 mM diamide for 4 h led to a release of protein and disruption of the parasites.
(TIF)

Figure S2 **The autofluorescence of red blood cells does not disturb the ratiometric measurement of hGrx1-roGFP2 in** ***Plasmodium falciparum*** **.** Comparison of mean fluorescence values of red blood cells (RBCs) and *P. falciparum* 3D7-infected red blood cells (iRBCs) containing the hGrx1-roGFP2 redox sensor by confocal microscopy. (**A–D**) Representative fluorescence measurement of RBC (purple) and iRBC (green) within a defined region of interest (ROI). (**A**) 405 nm (**B**) 488 nm (**C**, **D**) DIC. (**E**) 405/488 nm ratios of iRBCs, RBCs, and the mean fluorescence ratios of iRBCs subtracted from the ratios of RBCs (n = 3). (**F**) Mean fluorescence of iRBCs and RBCs normalized to 100% fluorescence of iRBCs at 405 nm excitation.
(TIF)

Figure S3 **hGrx1-roGFP2 enables live cell imaging of oxidation by diamide.** Different concentrations of diamide (∼0 to 1 mM) were evaluated to determine the concentration-dependent oxidation of the parasite cytosol. Diamide treatment was started after 60 s, and parasites were monitored for 4 min. 1 mM of diamide was constantly found to induce maximal oxidation. Merged (405/488 nm) images of the different concentrations at different time points are depicted. The ratio of emissions after excitation at 405 and 488 nm was computed and plotted against time. For each concentration, the data from 3 trophozoites was analyzed. Results are shown for the parasite strains 3D7 (**A–C**) and Dd2 (**D–F**).
(TIF)

Figure S4 **hGrx1-roGFP2 in different developmental stages of** ***P. falciparum*.** Schizont (**A**) and gametocyte stages (**B**) of *P. falciparum* parasites (here shown for the 3D7 strain) expressing hGrx1-roGFP2 were treated (after 60 s baseline monitoring) with 1 mM diamide and monitored for 4 min. 405 nm, 488 nm, and merged (405/488 nm) images at different time points are shown and indicate oxidation of the cytosol. (**C**) Reduction of trophozoite stages of *P. falciparum*. After 60 s, trophozoite stage parasites (here shown for the 3D7 strain) expressing hGrx1-roGFP2 were treated with 10 mM DTT and monitored for 4 min. The ratio (405/488 nm) images in the bottom line indicate reduction of the cytosol.
(TIF)

Figure S5 ***In vitro*** **interaction of the hGrx1-roGFP2 protein with redox-active compounds.** **A**. Dose response curves of diamide, GSSG, and H_2_O_2_ after 5 min incubation with recombinant hGrx1-roGFP2. **B–F**. Excitation spectra of hGrx1-roGFP2 in the presence of different concentrations of diamide, GSSG, H_2_O_2_, SIN1, and AAPH.
(TIF)

Figure S6 **Monitoring short-term oxidative effects of BSO and artemisinin derivatives with hGrx1-roGFP2.** After 60 s preincubation, the parasites were treated with 1 mM buthionine sulfoximine (BSO, **A**) or 100 µM artemisinin (ART, **B**), artemether (ATM, **C**), or artesunate (ATS, **D**) and monitored for 4 min. Merged (405/488 nm) and false color ratio images at different time points are shown.
(TIF)

Figure S7 **Monitoring short-term oxidative effects of quinoline antimalarial drugs with hGrx1-roGFP2.** After 60 s preincubation, the parasites were treated with 100 µM chloroquine (CQ, **A**), amodiaquine (AQ, **B**), quinine (QN, **C**), or mefloquine (MQ, **D**) and monitored for 4 min. Merged (405/488 nm) and false color ratio images at different time points are depicted.
(TIF)

Figure S8 **The redox state of** ***P. falciparum*** **thioredoxin after drug treatment.** (**A**) Western blot of recombinant *P. falciparum* thioredoxin 1 (PfTrx1) reduction with 5 mM DTT and with or without treatment of 10 mM iodoacetic acid. (**B**) Western blot of *P. falciparum* 3D7 Trx after incubation with 1 mM diamide, 4×IC_50_ of methylene blue (MB), or artemisinin (ART) for 24 h, or with 1 mM diamide, 50 mM H_2_O_2_, or 100×IC_50_ of MB for 15 min. (**C**) Western blot of *P. falciparum* 3D7 Trx after incubation with 10 µM, 50 µM, 100 µM, or 500 µM diamide for 24 h. Incubation with 1 mM and 500 µM diamide for 24 h led to complete disruption of the parasites. Incubation with 1 mM diamide for 15 min led to a partial disruption of the parasites.
(TIF)
