# Supplementary figures and images for: Real-Time Imaging of the Intracellular Glutathione Redox Potential in the Malaria Parasite Plasmodium falciparum
Source: PLoS Pathog. 2013 Dec 5;9(12):e1003782. doi: 10.1371/journal.ppat.1003782 (PMC3857819; doi:10.1371/journal.ppat.1003782)

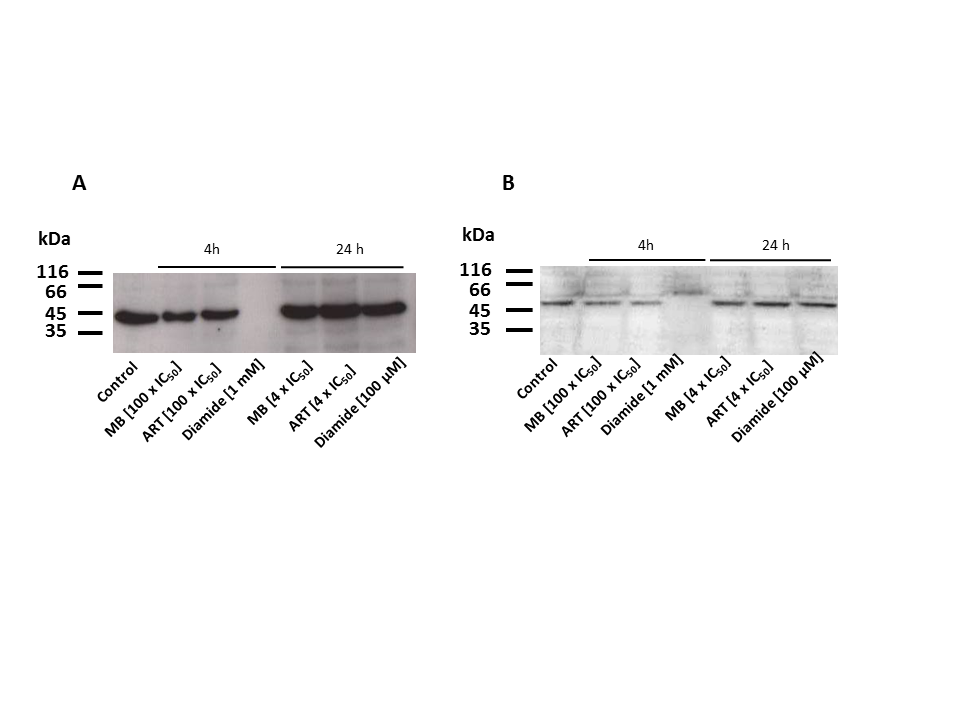

Supplement: Figure S1 — The redox sensor Grx-roGFP in P. falciparum remains intact after drug treatment. Western blots of Grx-roGFP transfected P. falciparum 3D7 with (A) anti-GFP and (B) anti-Grx antibody after incubation with different concentrations of methylene blue (MB), artemisinin (ART), or diamide for 4 or 24 h. Incubation with 1 mM diamide for 4 h led to a release of protein and disruption of the parasites. (TIF) [file ppat.1003782.s001.tif]

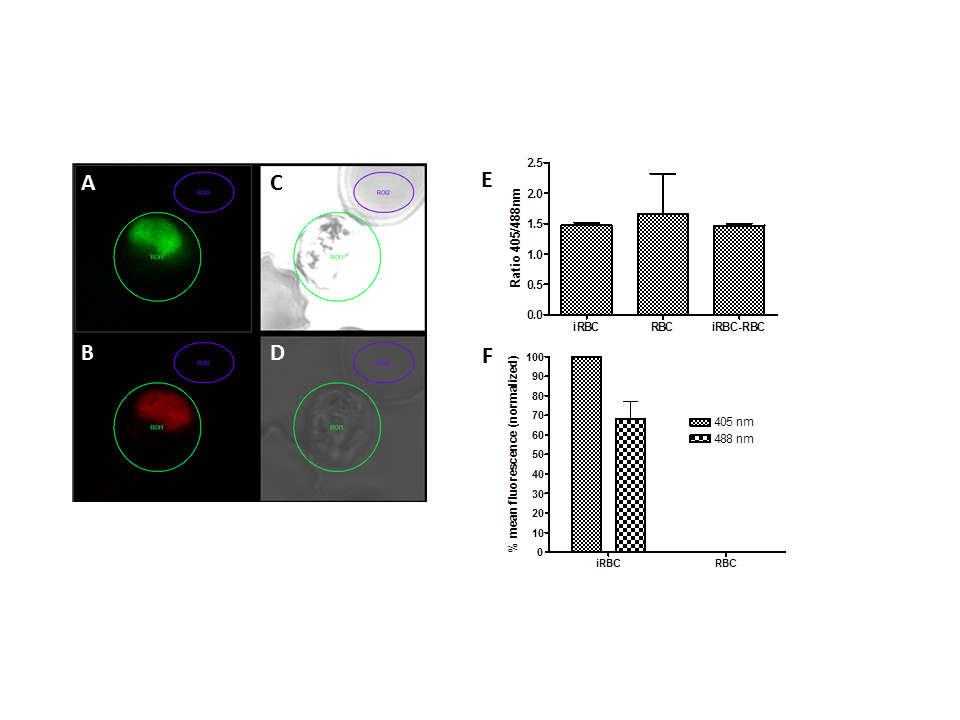

Supplement: Figure S2 — The autofluorescence of red blood cells does not disturb the ratiometric measurement of hGrx1-roGFP2 in Plasmodium falciparum . Comparison of mean fluorescence values of red blood cells (RBCs) and P. falciparum 3D7-infected red blood cells (iRBCs) containing the hGrx1-roGFP2 redox sensor by confocal microscopy. (A–D) Representative fluorescence measurement of RBC (purple) and iRBC (green) within a defined region of interest (ROI). (A) 405 nm (B) 488 nm (C, D) DIC. (E) 405/488 nm ratios of iRBCs, RBCs, and the mean fluorescence ratios of iRBCs subtracted from the ratios of RBCs (n = 3). (F) Mean fluorescence of iRBCs and RBCs normalized to 100% fluorescence of iRBCs at 405 nm excitation. (TIF) [file ppat.1003782.s002.tif]

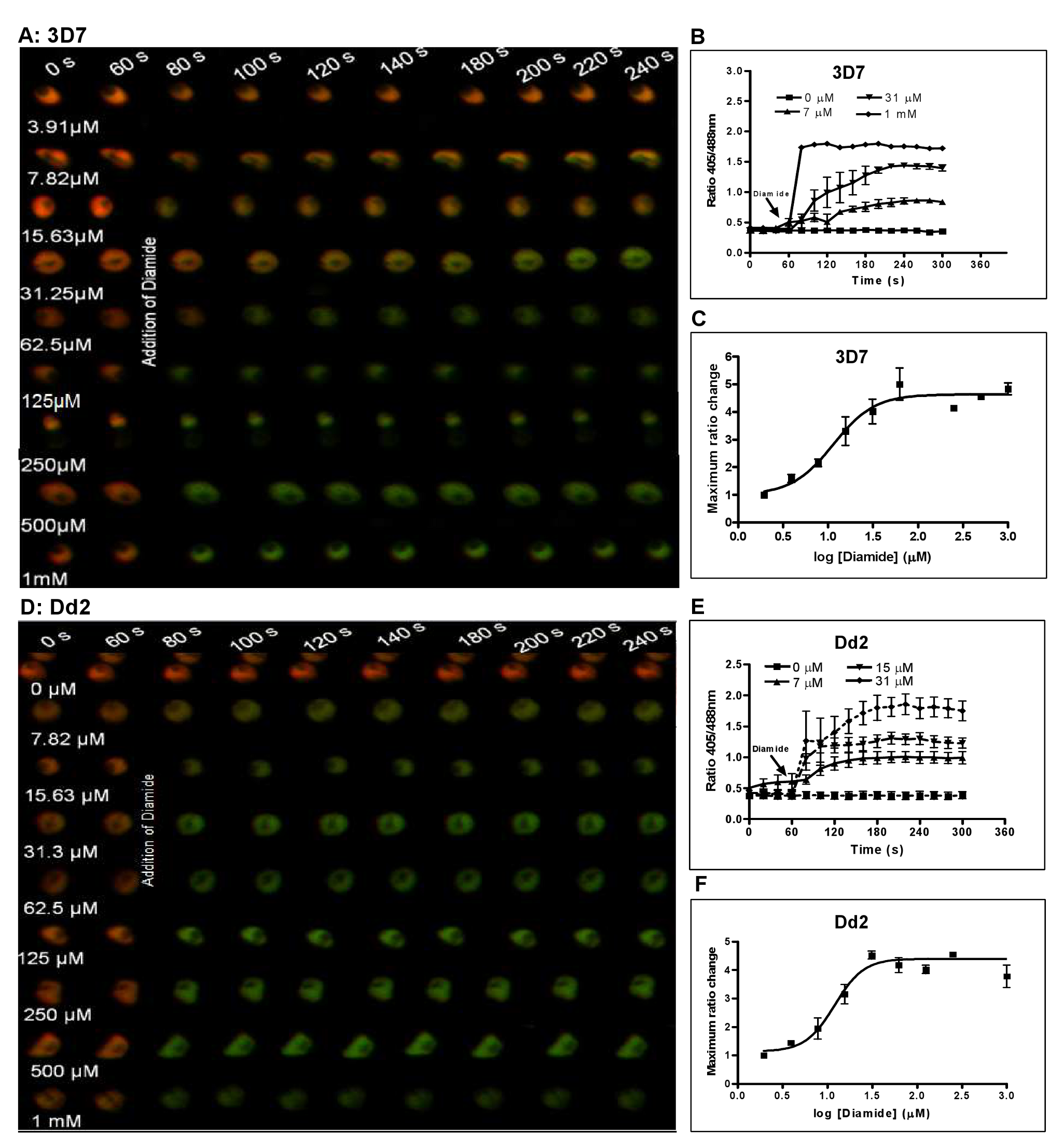

Supplement: Figure S3 — hGrx1-roGFP2 enables live cell imaging of oxidation by diamide. Different concentrations of diamide (∼0 to 1 mM) were evaluated to determine the concentration-dependent oxidation of the parasite cytosol. Diamide treatment was started after 60 s, and parasites were monitored for 4 min. 1 mM of diamide was constantly found to induce maximal oxidation. Merged (405/488 nm) images of the different concentrations at different time points are depicted. The ratio of emissions after excitation at 405 and 488 nm was computed and plotted against time. For each concentration, the data from 3 trophozoites was analyzed. Results are shown for the parasite strains 3D7 (A–C) and Dd2 (D–F). (TIF) [file ppat.1003782.s003.tif]

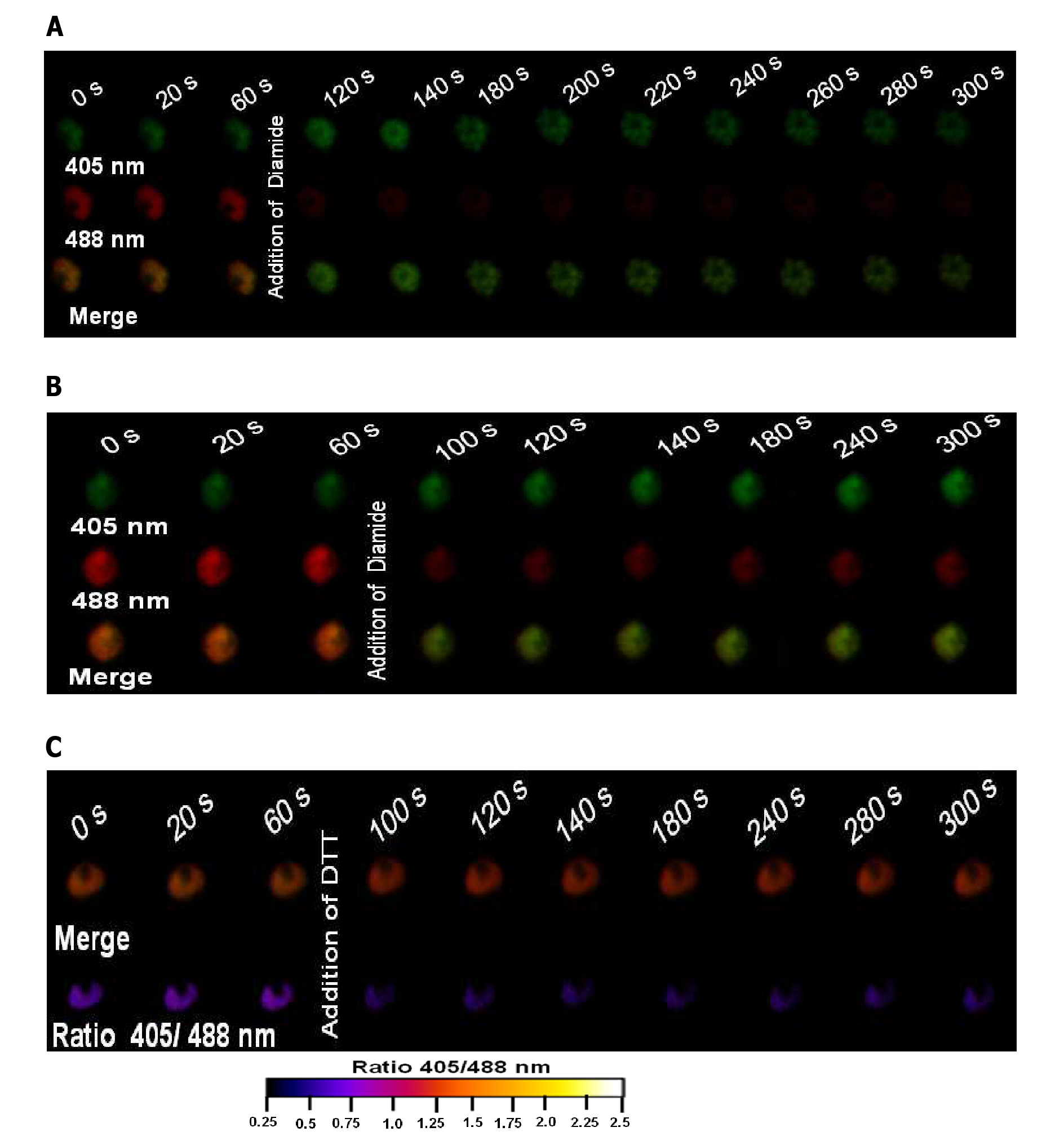

Supplement: Figure S4 — hGrx1-roGFP2 in different developmental stages of P. falciparum. Schizont (A) and gametocyte stages (B) of P. falciparum parasites (here shown for the 3D7 strain) expressing hGrx1-roGFP2 were treated (after 60 s baseline monitoring) with 1 mM diamide and monitored for 4 min. 405 nm, 488 nm, and merged (405/488 nm) images at different time points are shown and indicate oxidation of the cytosol. (C) Reduction of trophozoite stages of P. falciparum. After 60 s, trophozoite stage parasites (here shown for the 3D7 strain) expressing hGrx1-roGFP2 were treated with 10 mM DTT and monitored for 4 min. The ratio (405/488 nm) images in the bottom line indicate reduction of the cytosol. (TIF) [file ppat.1003782.s004.tif]

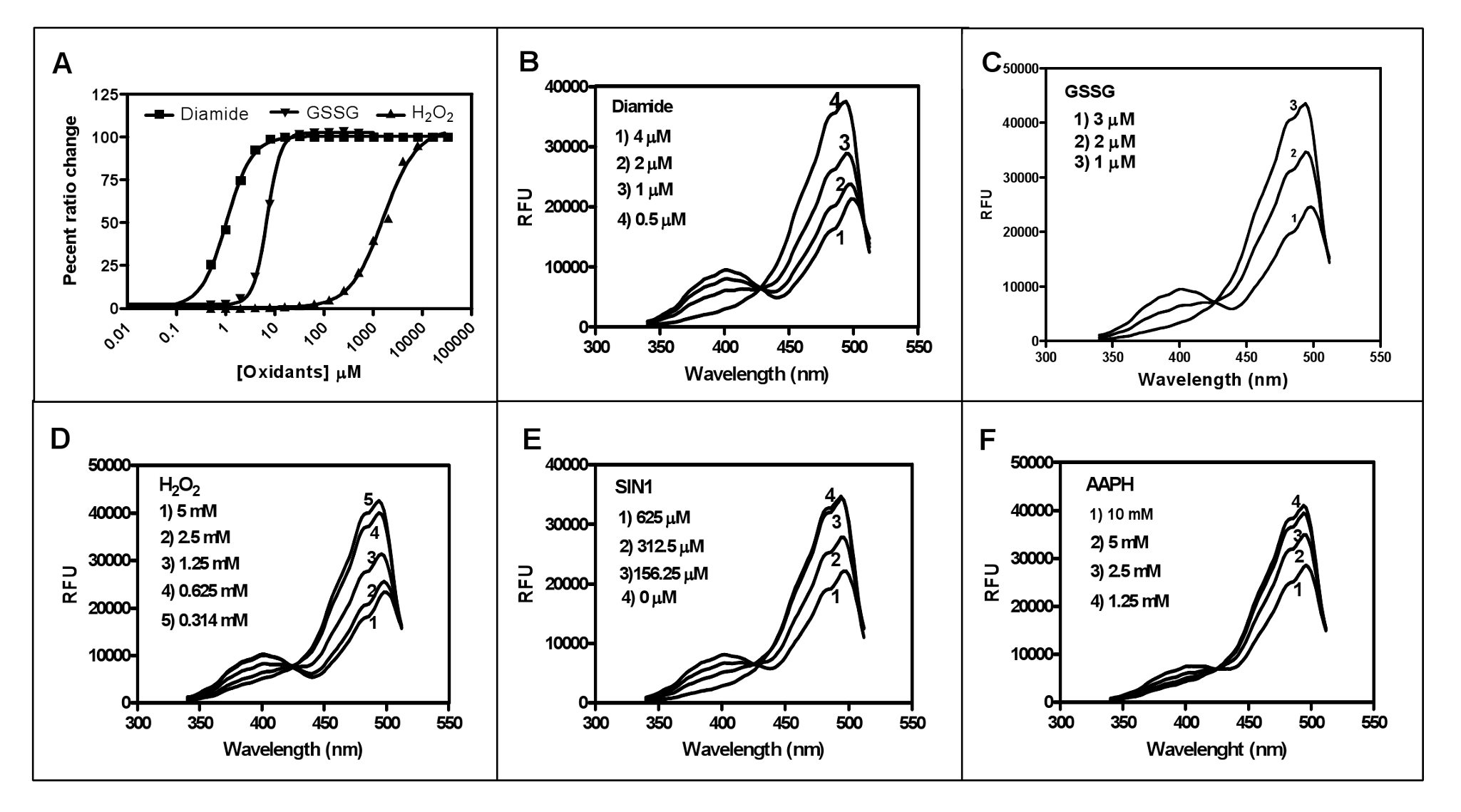

Supplement: Figure S5 — In vitro interaction of the hGrx1-roGFP2 protein with redox-active compounds. A. Dose response curves of diamide, GSSG, and H2O2 after 5 min incubation with recombinant hGrx1-roGFP2. B–F. Excitation spectra of hGrx1-roGFP2 in the presence of different concentrations of diamide, GSSG, H2O2, SIN1, and AAPH. (TIF) [file ppat.1003782.s005.tif]

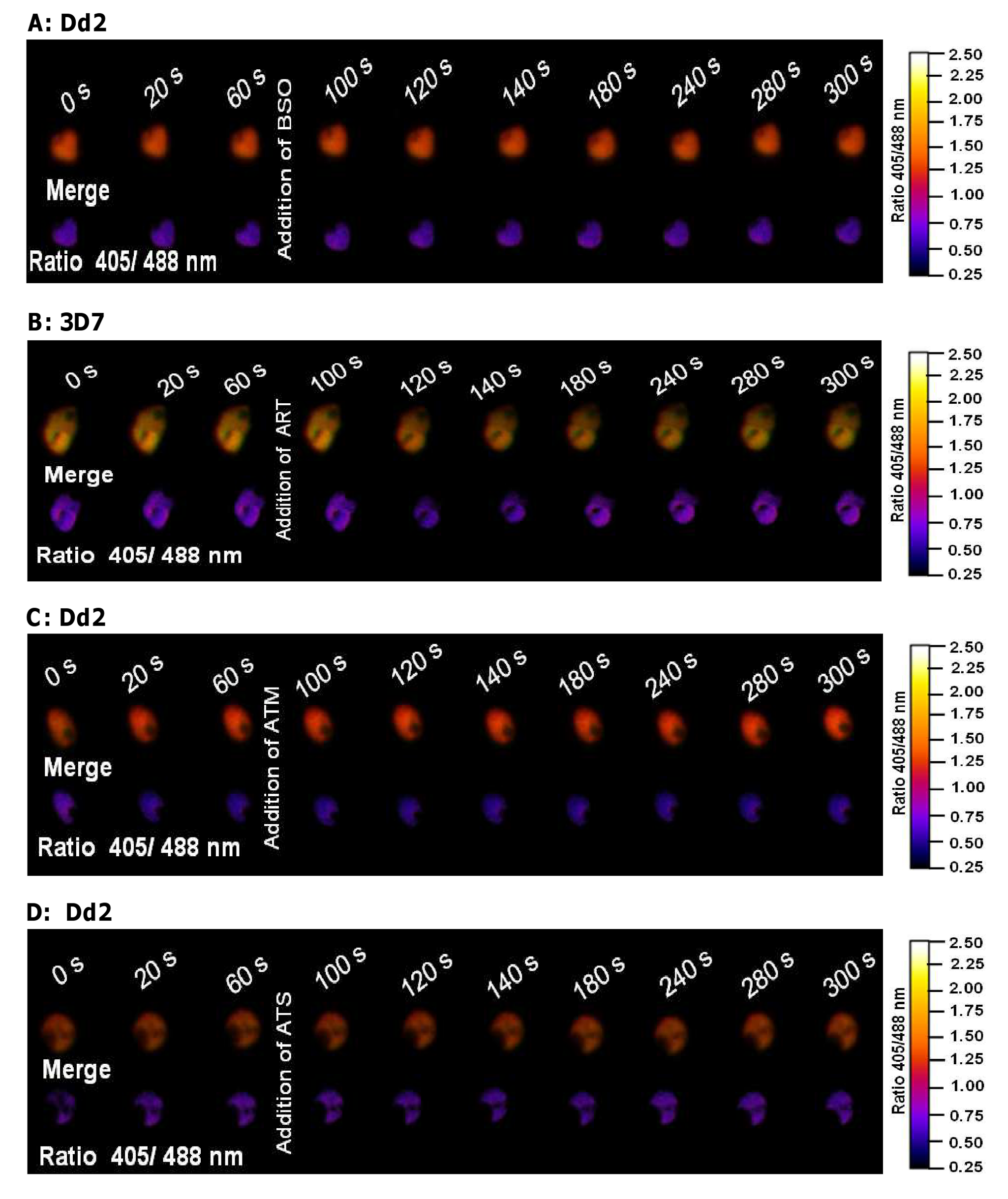

Supplement: Figure S6 — Monitoring short-term oxidative effects of BSO and artemisinin derivatives with hGrx1-roGFP2. After 60 s preincubation, the parasites were treated with 1 mM buthionine sulfoximine (BSO, A) or 100 µM artemisinin (ART, B), artemether (ATM, C), or artesunate (ATS, D) and monitored for 4 min. Merged (405/488 nm) and false color ratio images at different time points are shown. (TIF) [file ppat.1003782.s006.tif]

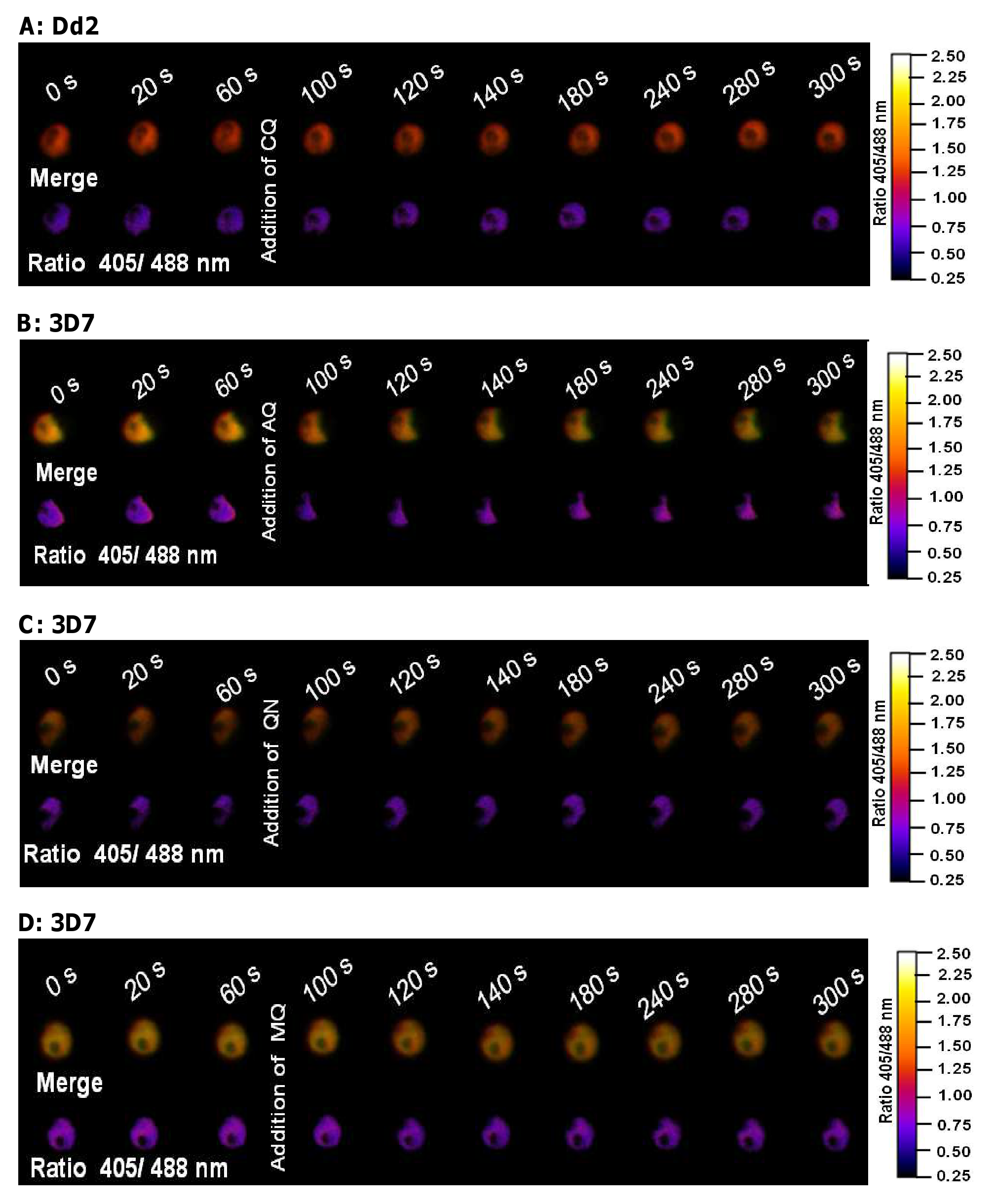

Supplement: Figure S7 — Monitoring short-term oxidative effects of quinoline antimalarial drugs with hGrx1-roGFP2. After 60 s preincubation, the parasites were treated with 100 µM chloroquine (CQ, A), amodiaquine (AQ, B), quinine (QN, C), or mefloquine (MQ, D) and monitored for 4 min. Merged (405/488 nm) and false color ratio images at different time points are depicted. (TIF) [file ppat.1003782.s007.tif]
